# Argentophilic Interactions, Flexibility, and Dynamics of Pyrrole Cages Encapsulating Silver(I) Clusters

**DOI:** 10.1021/acs.jpca.4c01464

**Published:** 2024-04-23

**Authors:** Bartosz Trzaskowski, Juan Pablo Martínez, Aleksandra Sarwa, Bartosz Szyszko, William A. Goddard

**Affiliations:** † Centre of New Technologies, University of Warsaw, 2C Banacha Street, 02-097 Warszawa, Poland; ‡ Faculty of Chemistry, University of Wrocław, 14 F. Joliot-Curie Street, 50-387 Wrocław, Poland; § Materials and Process Simulation Center, California Institute of Technology, Pasadena, California 91106, United States

## Abstract

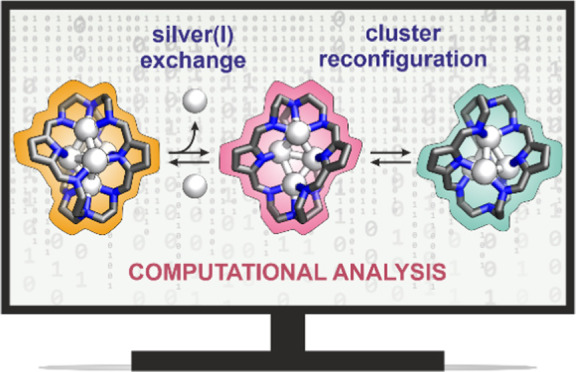

Recently, pyrrole cages have been synthesized that encapsulate ion pairs and silver(I) clusters to form intricate supramolecular capsules. We report here a computational analysis of these structures using density functional theory combined with a semiempirical tight-binding approach. We find that for neutral pyrrole cages, the Gibbs free energies of formation provide reliable predictions for the ratio of bound ions. For charged pyrrole cages, we find strong argentophilic interactions between Ag ions on the basis of the calculated bond indices and molecular orbitals. For the cage with the Ag_4_ cluster, we find two minimum-geometry conformations that differ by only 6.5 kcal/mol, with an energy barrier <1 kcal/mol, suggesting a very flexible structure as indicated by molecular dynamics. The predicted energies of formation of **[Ag***_**n**_*⊂**1]**^***n*-3+**^ (*n* = 1–5) cryptands provide low energy barriers of formation of 5–20 kcal/mol for all cases, which is consistent with the experimental data. Furthermore, we also examined the structural variability of mixed-valence silver clusters to test whether additional geometrical conformations inside the organic cage are thermodynamically accessible. In this context, we show that the time-dependent density functional theory UV–vis spectra may potentially serve as a diagnostic probe to characterize mixed-valence and geometrical configurations of silver clusters encapsulated into cryptands.

## Introduction

Subcomponent self-assembly is a robust synthetic methodology that employs the formation of covalent (C=N) and coordinative (N → M) bonds to generate complex three-dimensional discrete supramolecular architectures in multicomponent reactions that provide products of high symmetry and complexity.^1^ Exploiting self-assembly methodology offers an opportunity to build extensive metallosupramolecular architectures from simple organic building blocks and metals. This synthetic approach has allowed the construction of intricate architectures, including helicates,^2^ mechanically interlocked molecules,^3,4^ and functional capsules.^5^

Molecular cages are three-dimensional molecules with well-defined internal cavities.^6,7^ The design of intricate supramolecular capsules, initially driven by their unique structures and aesthetic appeal, led to the discovery of their remarkable properties and functions, stimulating developments in supramolecular chemistry. Metal–organic capsules demonstrate numerous intriguing features that allow their exploitation in separating small molecules,^5^ sensing,^8^ metal nanoparticle generation,^9^ drug delivery,^10^ and anion transport.^11^ The tailored voids of molecular capsules, protected from the surrounding, allow entrapment of reactive species^12^ which was exploited to perform catalytic transformations that would be unattainable under typical reaction conditions.^13^ This capsule cavity design allows formation of intramolecular interactions between the host and the guest that lead to selective incorporation and release of the molecular cargo.^14^

The tris(2-aminoethyl)amine (tren)-based imine aza cryptands developed by Martell and Lehn,^15−19^ Pascard,^20^ and Nelson^21−25^ can be considered archetypical imine-based cage systems. These molecules are typically synthesized using Schiff condensation between tren and a respective aldehyde to introduce the functional motif into the molecule. The choice of spacers fundamentally alters the properties and coordination preferences of the cage and their affinity toward guests. Tren-based cryptands were demonstrated to act as effective anion receptors after transformation into mono- or binuclear metal complexes. Upon anion encapsulation, they formed cascade cryptates,^16,26,27^ in which the anionic entity is entrapped by two metal centers coordinated within the trenimine sites.

**Scheme 1:**
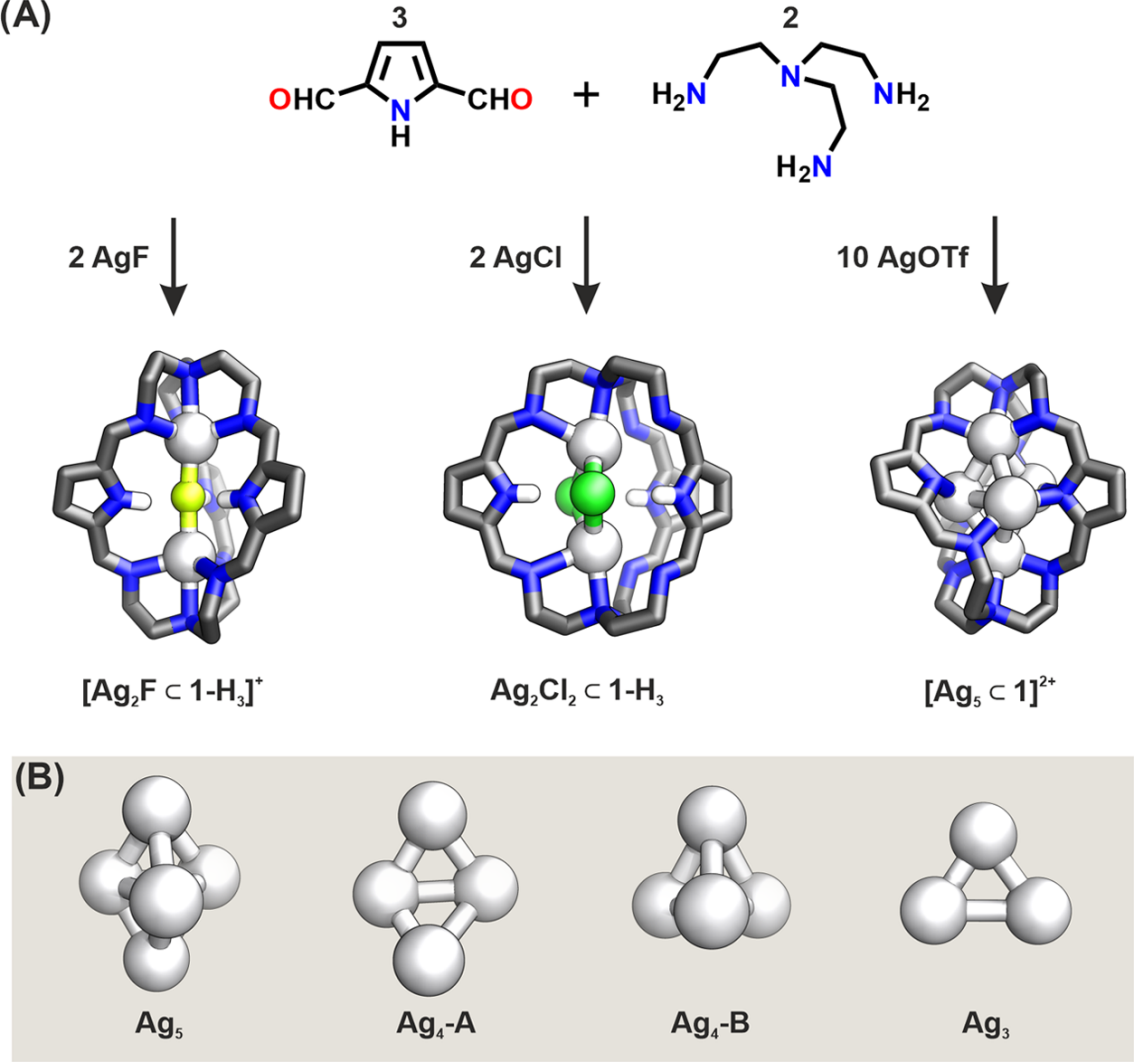
(A) Synthesis of Silver(I) Cryptates and (B) Geometries of the Observed Silver(I) Clusters^29^

A particularly intriguing property of cryptates is their coordination plasticity—the ability of metal cations to undergo rearrangements within the cavity, requiring in some cases exchange with cations outside the capsule.^28^ This unique mode of reactivity was illustrated by Nelson, who described the transformation of the disilver cage into the trisilver cryptate, enabling both modification of metal coordination sites within the cage and also reconfiguration of the capsule conformation.^28^ Recently, we reported on the iminopyrrole cage,^29^ which, depending on the silver(I) source, formed cascade cryptates embedding fluoride or chloride between two metal centers, or else formed plenates, i.e., cryptates incorporating silver(I) clusters accommodated within the cavity (Scheme 1).^29^

Quantum chemical computational methodologies serve as predictive approaches to determine cage structure, topology, cavity size, and associated molecular properties within extensive data sets of porous organic cages.^30^ This enables the guidance of synthetic researchers toward the identification and development of materials possessing targeted properties. For example, the study conducted by Jelfs et al. focused on the predictive modeling of shape persistence in organic cages.^31^ In their research, a comprehensive data set consisting of 63,472 organic cages with diverse topologies was meticulously assembled using an automated computational approach. This involved the synthesis of organic cages through chemical reactions from diverse building blocks. The results revealed that cages formed by imine condensation of trialdehydes and diamines in a [4 + 6] reaction exhibited the highest probability of shape persistence, while thiol reactions were more prone to yield dissociated cages. Using this extensive library and employing machine learning models, the authors achieved a remarkable accuracy of up to 93% in predicting shape persistence. Furthermore, evolutionary algorithms offer a means to determine the necessary dimensions of precursor molecules for achieving a predetermined cage cavity size and associated properties.^32^ Therefore, the convergence of computational and experimental methodologies holds great promise for accelerating the discovery of new cage systems.^33,34^ Particularly noteworthy is the application of rapid screening techniques of organic cages, thereby offering a consistent methodology to facilitate the development of materials with precisely tailored characteristics,^35,36^ as well as the use of time-dependent DFT approaches to obtain detailed information about the electronic structure of metal clusters inside cages.^37,38^ Here, we provide a detailed computational analysis of the recently described iminopyrrole cages, focusing on their intracavity dynamics, coordination plasticity, and other important properties, for which computational techniques can provide data complementary to the experimental data. Apart from the already mentioned plasticity of the system, we pay particular attention to the properties of potential mixed-valence Ag clusters, as this topic has recently attracted significant attention in the context of luminescence,^39^ fluorescence,^40^ chemical reactivity,^41,42^ and other applications.^43^

## Methods

In all calculations, the starting structures were based on the available crystal structures of pyrrole cages encapsulating selected ions.^29^ Most calculations were performed using a two-step approach. First, the geometries of the starting structures were optimized at the GFN2-xTB level of theory (as implemented in the xtb ver. 6.4.1 software) with the analytical linearized Poisson–Boltzmann model of acetonitrile.^44,45^ Second, structures optimized in the GFN2-xTB method were subjected to density functional calculations using the wB97X-d functional with the 6-31G** basis set on all atoms except Ag, which was described using the LANL2DZ ECP basis set, as implemented in the Gaussian 16 software.^46−53^ For comparison, we also performed selected calculations using the same functional and the 6-31G** basis set on all light atoms but with the WTBS all-electron basis set for Ag.^54−57^

The Gibbs free energies were defined as the sum of the electronic energy, zero-point energy correction, and thermal corrections to Gibbs free energy, including the entropy term, all at 298.15 K. The Gibbs free energies of solvation were estimated using the SMD solvation model for acetonitrile, as implemented in Gaussian 16,^53^ and added to the final Gibbs free energies.^58^ p*K*_a_ calculations were performed using a density functional-based approach implemented in Jaguar ver. 11.2^59^ using the standard thermodynamic cycle for acid dissociation in both the water and gas phases.^60^ Conformational searches were performed using the Conformer-Rotamer Ensemble Sampling Tool (CREST) ver. 2.12 at the GFN2-xTB level of theory.^61^ The values of interaction energies/Gibbs free energies include the counterpoise correction to the basis set superposition error (BSSE).^62^ The selected methodology aligns with the best-practice DFT protocols as delineated by Grimme et al.^63^ In this context, (i) we performed initial conformational screenings; (ii) geometry optimizations and thermochemistry were evaluated via an accurate DFT method (wB97X-d); and (iii) examined BSSE and basis set incompleteness error. Furthermore, the wB97X-d functional has been utilized in the evaluation of argentophilic interactions in aza cryptands encapsulating a silver(I) cation to interpret experimental results.^64^ Additionally, we also used symmetry-adapted perturbation theory SAPT0 approach^65,66^ with the def2-svp^67−69^ basis set (as implemented in Psi4 1.6.1 software^70^) to accurately describe interactions and binding energies. Bond indices and partial charges were analyzed using the Multiwfn ver. 3.7 program.^71^ Figures were prepared using VMD software ver. 1.9.3^72^ and xyzviewer software written by Sven de Marothy.^73^

## Results and Discussion

### Metal-Free Cages

**Figure 1:**
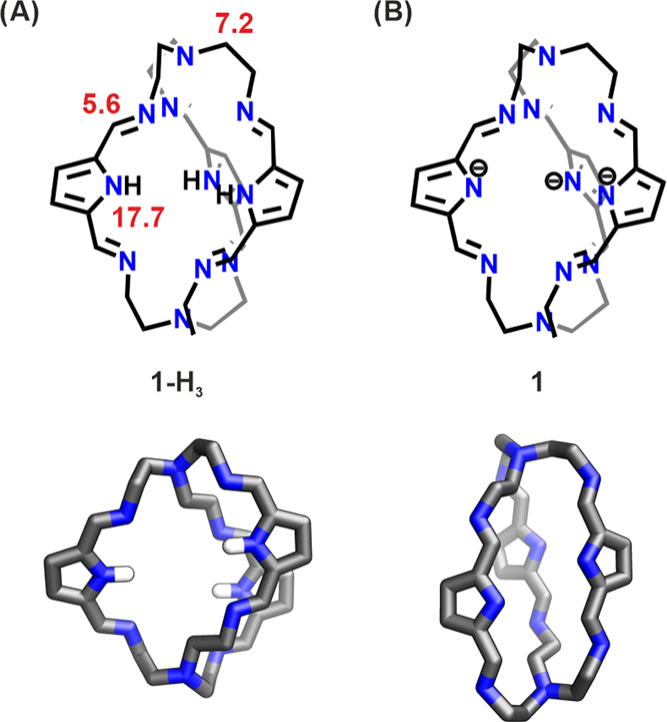
Protonated (**1-H**_**3**_) and deprotonated (**1**^**3–**^) forms of the pyrrole cage compared with DFT-optimized structures (the lowest-energy conformers) of these cages. The average p*K*_a_ values of the amine, imine, and pyrrole N atoms are depicted in red.

In the initial stage, we considered the metal-free pyrrole cages, namely, the neutral **1-H**_**3**_ and trianionic **1**^**3–**^ incorporating pyrrolide ligands formed upon formal deprotonation of **1-H**_**3**_ (Figure 1). Here, we carried out a conformational search at the GFN2-xTB level of theory separately for **1**^**3–**^ and **1-H**_**3**_ and then performed complete DFT geometry optimization of the five lowest-energy conformers for each case. In the case of **1-H**_**3**_, we found four conformations within the 0.4 kcal/mol window, which is within the accuracy of the DFT approach. In the case of **1**^**3–**^, one conformation is far lower (more than 7 kcal/mol) in Gibbs free energy than all other conformations (see Table S1). These minimum-energy conformations were used to estimate the strain energy of the cages, defined as the difference between the energy of the cage conformation when it encapsulates the silver clusters and the energy of the lowest-energy apo conformer. As expected, the strain energies are moderate for the neutral cage **1-H**_**3**_ (20–40 kcal/mol) but relatively large for **1**^**3–**^ (70–100 kcal/mol) because the negatively charged entity drastically changes its conformation after encapsulating silver ions. In particular, the neutral **1-H**_**3**_ adopted the conformation with all three pyrrole NH groups pointing into the center of the cavity, whereas upon deprotonation, the molecule rearranged in a way that the pyrrolide and imine nitrogen atoms faced outside the cavity. This arrangement is likely to minimize the repulsion of the electron density centered on the nitrogen atoms and to increase the interaction with the polar solvent molecules.

For the pyrrole cage, we also estimated the p*K*_a_ values of all nitrogen atoms. The average p*K*_a_ value of the three pyrrole N atoms is 17.7, close to the experimental p*K*_a_ value of pyrrole of 17.5. The average value of the p*K*_a_ of the six imine N atoms is equal to 5.6, which is within the range of p*K*_a_ values of imine N atoms (5–7). Finally, the average value of the p*K*_a_ of the two tertiary N atoms is 7.2, approximately 2.5 pH units lower than that of trimethylamine. These values serve as indicators of the propensity of the N atoms to be protonated, thus enabling the prediction of structural modifications via acid–base reactions, particularly in the formation of the conjugate base pyrrolide that yields the trianionic structure **1**^**3–**^.

### Cascade Cryptates

**Figure 2:**
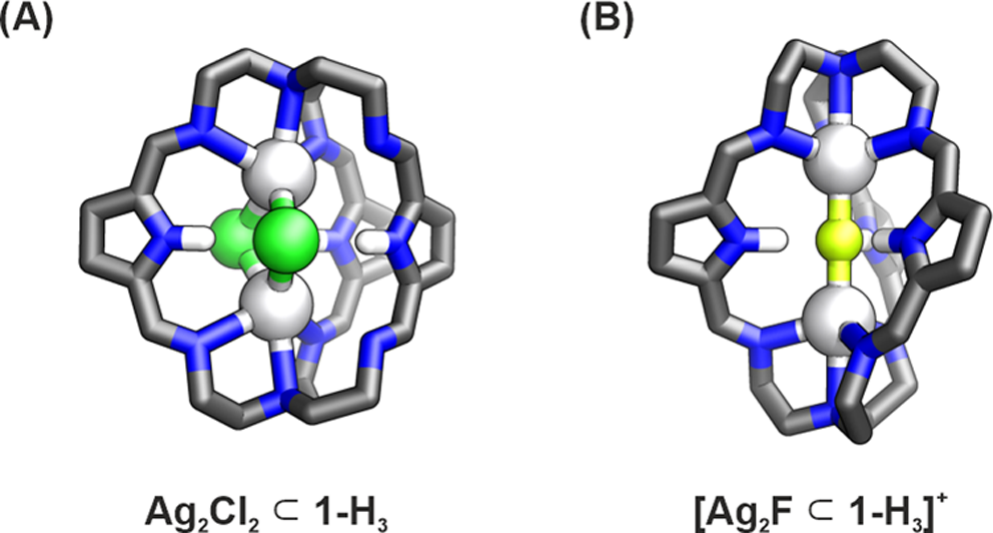
DFT-optimized structures of the protonated form of the pyrrole cage encapsulating (A) Ag_2_Cl_2_ and (B) Ag_2_F.

For the previously synthesized cascade cryptates **Ag**_**2**_**Cl**_**2**_⊂**1-H**_**3**_ and **[Ag**_**2**_**F**⊂**1-H**_**3**_**]**^**+**^ (Figure 2), we estimated Gibbs free energies of the following hypothetical reactions:
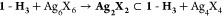
1
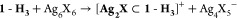
2We then constructed and optimized similar complexes **Ag**_**2**_**F**_**2**_⊂**1-H**_**3**_ and **[Ag**_**2**_**Cl**⊂**1-H**_**3**_**]**^**+**^ as well as both combinations of **Ag**_**2**_**X**_**2**_⊂**1-H**_**3**_ and **[Ag**_**2**_**X**⊂**1-H**_**3**_**]**^**+**^, where X = Br or I. The results in Table 1 agree with the experimental trends showing that the Δ*G* of **Ag**_**2**_**Cl**_**2**_⊂**1-H**_**3**_ formation is lower by 6.8 kcal/mol than that of **[Ag**_**2**_**Cl**⊂**1-H**_**3**_**]**^**+**^ and, vice versa, the Δ*G* of formation of **[Ag**_**2**_**F**⊂**1-H**_**3**_**]**^**+**^ is lower (more favorable by 3 kcal/mol) than that of **Ag**_**2**_**F**_**2**_⊂**1-H**_**3**_. Based on these results, we predict that both Br^–^ and I^–^ ions will preferably form **Ag**_**2**_**X**_**2**_⊂**1-H**_**3**_-type cryptands, rather than **[Ag**_**2**_**X**⊂**1-H**_**3**_**]**^**+**^ with geometries very similar to the **Ag**_**2**_**Cl**_**2**_⊂**1-H**_**3**_ case (although in the case of I^–^ both Δ*G* values are very similar and within the expected accuracy of the DFT approach). This result is intuitive, as the increasing ionic radius of bromide and iodide is expected to limit the ability of these anions to incorporate between two silver(I) centers. Unfortunately, the experimental results did not allow verification of these findings because the attempted bromide- and iodide-incorporating cascade cryptates were prone to decomposition and could not be isolated.

**Table 1:** Interaction and Strain Energies, Δ*G*, of Binding between the Ag_2_X_2_/Ag_2_X^+^ and **1-H**_**3**_, and Gibbs Free Energies of Formation of Cryptands

system	DFT interaction energy (kcal/mol)	SAPT0 interaction energy (kcal/mol)	strain energy (kcal/mol)	Δ*G* of binding (kcal/mol)	Δ*G* of formation (kcal/mol)
**Ag**_**2**_**F**_**2**_⊂**1-H**_**3**_	–135.6	–119.6	54.6	–79.8	–63.8
**Ag**_**2**_**Cl**_**2**_⊂**1-H**_**3**_	–95.9	–79.7	28.3	–49.8	–46.0
**Ag**_**2**_**Br**_**2**_⊂**1-H**_**3**_	–88.9	–75.9	25.9	–43.0	–37.8
**Ag**_**2**_**I**_**2**_⊂**1-H**_**3**_	–81.8	–70.8	23.0	–44.6	–36.0
**[Ag**_**2**_**F**⊂**1-H**_**3**_**]**^**+**^	–180.0	–156.3	38.0	–123.8	–66.8
**[Ag**_**2**_**Cl**⊂**1-H**_**3**_**]**^**+**^	–158.1	–138.6	36.2	–66.5	–39.2
**[Ag**_**2**_**Br**⊂**1-H**_**3**_**]**^**+**^	–150.4	–133.4	34.0	–60.2	–26.8
**[Ag**_**2**_**I**⊂**1-H**_**3**_**]**^**+**^	–154.1	–139.6	33.8	–63.3	–35.8

The interaction energies (Δ*E*), structural-strain energy (Δ*E*_str_), and the Δ*G* of binding between the Ag_2_X_2_/Ag_2_X^+^ and the pyrrole cage were estimated at the DFT level using the equations:
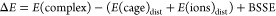
3
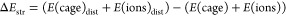
4
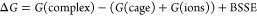
5where subscript dist stands for the distorted structure corresponding to the geometry of the complex (otherwise the geometry is optimized), BSSE is the basis set superposition error correction, and the values of Gibbs free energies are corrected for the solvation energy. The results shown in Table 1 reveal that anion size is of fundamental importance, with larger anions (Br^–^ and I^–^) having lower interaction energies with the cage in both the neutral and charged systems. This is particularly clear for I^–^ ion, where the optimized geometries of both **Ag**_**2**_**I**_**2**_⊂**1-H**_**3**_ and **[Ag**_**2**_**I**⊂**1-H**_**3**_**]**^**+**^ reveal that these ions are relatively far from the center of the cage. Nevertheless, for each halide, the Δ*E* and Δ*G* of binding are clearly more stabilizing for **[Ag**_**2**_**X**⊂**1-H**_**3**_**]**^**+**^. For example, the interactions Δ*E* between cage **1-H**_**3**_ and [Ag_2_F]^+^ in **[Ag**_**2**_**F**⊂**1-H**_**3**_**]**^**+**^ complex are 44.4 kcal/mol (DFT) or 36.7 kcal/mol (SAPT0) more stabilizing than the Δ*E* calculated for **Ag**_**2**_**F**_**2**_⊂**1-H**_**3**_. This trend is observed for each comparison of Δ*E* and Δ*G* values so that the strain energy becomes the determining factor that accounts for the predominant form of the complexes. That is, the Δ*E*_str_ associated with **[Ag**_**2**_**F**⊂**1-H**_**3**_**]**^**+**^ is 16.6 kcal/mol lower than that for **Ag**_**2**_**F**_**2**_⊂**1-H**_**3**_. Consequently, this decreased structural strain is reflected in a more favorable Δ*G* of formation for **[Ag**_**2**_**F**⊂**1-H**_**3**_**]**^**+**^. On the other hand, the predominant form in the case of chloride is **Ag**_**2**_**Cl**_**2**_⊂**1-H**_**3**_, a fact associated with the decreased Δ*E*_str_ and the favored Δ*G* of formation compared to the respective values for **[Ag**_**2**_**Cl**⊂**1-H**_**3**_**]**^**+**^. A similar observation can be made for the cryptates containing Br and I. Overall, Δ*E* and Δ*G* of binding account for the highly favored chemical insertion of the ions into the cavity of the cage, and Δ*E*_str_ and Δ*G* of formation serve as predictive tools of the predominant form of the complex.

**Figure 3:**
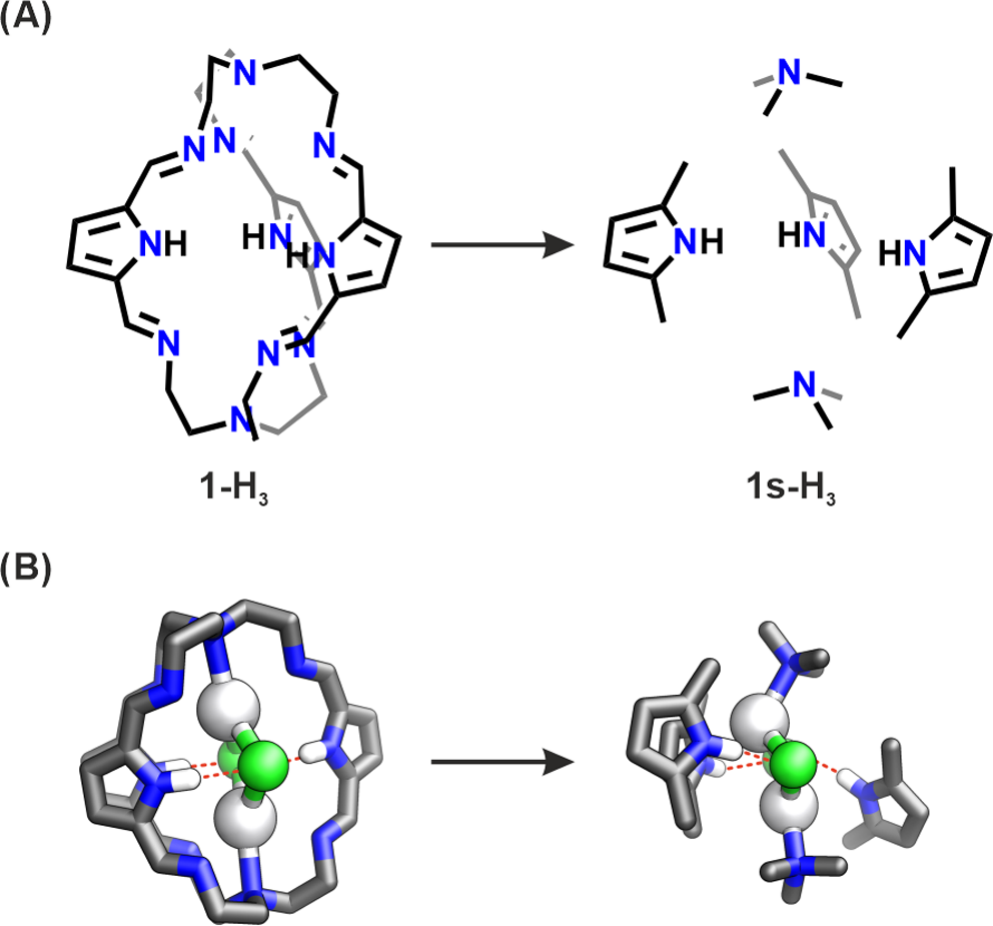
Structural reduction of the **1-H**_**3**_ cage in (A) a schematic representation and (B) optimized geometries of the **Ag**_**2**_**Cl**_**2**_⊂**1-H**_**3**_ and **Ag**_**2**_**Cl**_**2**_⊂**1s-H**_**3**_ systems highlighting the optimal geometries of the pyrrole and tertiary amine moieties.

The cascade cryptate **Ag**_**2**_**Cl**_**2**_⊂**1-H**_**3**_ was compared with its simplified model consisting of three 2,5-dimethylpyrrole units and two trimethylamine molecules (Figure 3). This system includes only minimal geometrical constraints, making it possible for all crucial atoms to attain the optimal geometry for interacting with the ions. Despite constituting a fair geometric representation, this model has limitations, resulting from the lack of imine nitrogen atoms, that play a vital role in the interactions with Ag^+^ ions and stabilization of the entire cluster. Even so, the results suggest that the positions of the five crucial N atoms in the **1-H**_**3**_ cage are close to ideal, since for the **Ag**_**2**_**Cl**_**2**_⊂**1-H**_**3**_ system, the value of the root-mean-square deviation (RMSD) between these atoms in the full-atom and the reduced system is 0.95 Å, while for **[Ag**_**2**_**F**⊂**1-H**_**3**_**]**^**+**^, it is 1.31 Å. Interestingly, the interaction energies between the cage and the ions in these simplified systems are smaller than Δ*E* values for the original structures (−82.7 kcal/mol versus −95.9 kcal/mol for **Ag**_**2**_**Cl**_**2**_⊂**1-H**_**3**_ and −125.6 kcal/mol versus −180.0 kcal/mol for **[Ag**_**2**_**F**⊂**1-H**_**3**_**]**^**+**^), thereby showing that imine N atoms are also responsible for non-negligible interactions with ions.

**Figure 4:**
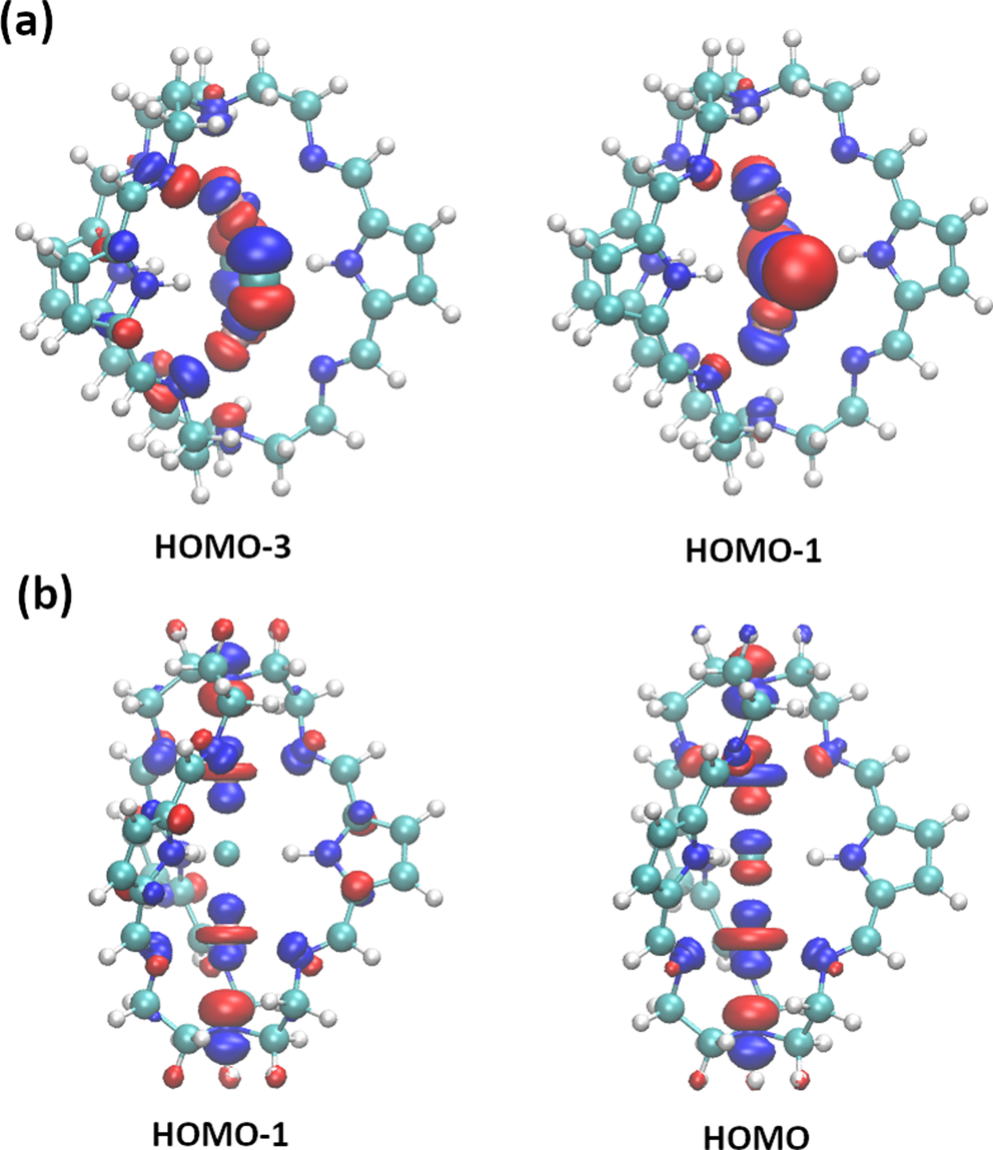
Selected frontier orbitals of (a) **Ag**_**2**_**Cl**_**2**_⊂**1-H**_**3**_ and (b) **[Ag**_**2**_**F**⊂**1-H**_**3**_**]**^**+**^.

We also analyzed the frontier orbitals of **Ag**_**2**_**Cl**_**2**_⊂**1-H**_**3**_ and **[Ag**_**2**_**F**⊂**1-H**_**3**_**]**^**+**^ (Figure 4), selected bond orders within these systems, including Wiberg, Mayer, and fuzzy-atomic-spaces aproaches,^74−76^ as well as selected partial charges (see Table 2 for selected values and Tables S2 and S3 for additional data), with a particular emphasis on potential argentophilic interactions. First, for both systems, we see at least two frontier orbitals having contributions from Ag and Cl orbitals, suggesting that there are interactions not only between the Ag^+^ and Cl^–^ ions but also between the ions and the cage and possibly also between the two Ag^+^ ions. The analysis of bond indices reveals a 0.10 Wiberg bond index (WBI) for Ag–Ag in **Ag**_**2**_**Cl**_**2**_⊂**1-H**_**3**_ and a negligible WBI for Ag–Ag in **[Ag**_**2**_**F**⊂**1-H**_**3**_**]**^**+**^; together with the visual inspection of frontier orbitals (Figure 4), these results suggests that interactions between the two Ag^+^ ions are favored by orbital interactions inducing electron delocalization rather than covalent bonding, as evidenced by the vanishing WBI values. In both systems, Ag^+^ ions also seem to form stronger interactions with imine nitrogen atoms (WBI between 0.30 and 0.45, Table S2) and slightly weaker interactions with amine nitrogen atoms at the cage poles (WBI of 0.27 in all cases). Identical values of bond indices for selected pairs of interactions revealed that both systems are highly symmetric. However, a detailed analysis of partial charges shows that **[Ag**_**2**_**F**⊂**1-H**_**3**_**]**^**+**^ is more symmetric than **Ag**_**2**_**Cl**_**2**_⊂**1-H**_**3**_ because all crucial partial charges are identical (see Tables 2 and S3).

**Table 2:** Selected Average Wiberg Bond Order Values and Partial Charges (in |e|) for **Ag**_**2**_**Cl**_**2**_⊂**1-H**_**3**_ and **[Ag**_**2**_**F**⊂**1-H**_**3**_**]**^**+**^

bond	Wiberg index	atom	Mulliken partial charge	natural partial charge
**Ag**_**2**_**Cl**_**2**_⊂**1-H**_**3**_				
Ag–Ag	0.10	Ag	0.14	0.51
Ag–Cl	0.55	Cl	–0.49	–0.71
Ag–N_amine_	0.27	N_amine_	–0.43	–0.55
Ag–N_imine_	0.25	N_imine_	–0.33	–0.47
**[Ag**_**2**_**F**⊂**1-H**_**3**_**]**^**+**^				
Ag–Ag	<0.05	Ag	0.20	0.57
Ag–F	0.45	F	–0.46	–0.68
Ag–N_amine_	0.27	N_amine_	–0.44	–0.56
Ag–N_imine_	0.45	N_imine_	–0.38	–0.52

### Pyrrolide Cages Incorporating Silver(I) Clusters

**Figure 5:**
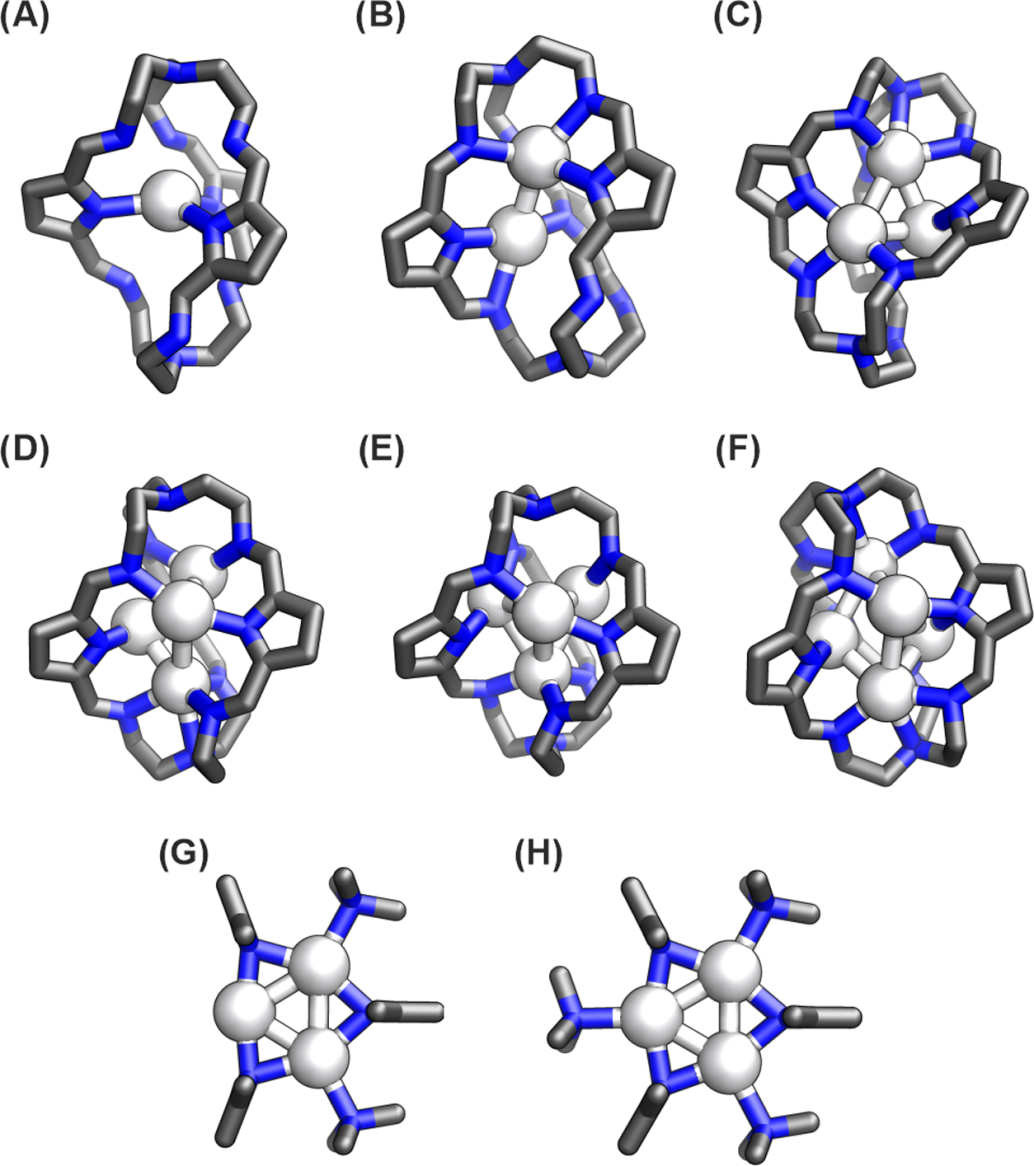
DFT-optimized structures of the deprotonated form of the pyrrole cage encapsulating silver cluster: (A) **[Ag**⊂**1]**^**2–**^, (B) **[Ag**_**2**_⊂**1]**^**–**^, (C) **Ag**_**3**_⊂**1**, (D) **[Ag**_**4**_**-A**⊂**1]**^**+**^, (E) **[Ag**_**4**_**-B**⊂**1]**^**+**^, and (F) **[Ag**_**5**_⊂**1]**^**2+**^; simplified complexes with Ag_3_ cluster: (G) three pyrrole and two trimethylamine moieties and (H) three pyrrole and three trimethylamine moieties.

For the silver(I) cluster-incorporating cages, **[Ag***_**n**_*⊂**1]**^***n*-3+**^, the interaction energies between the Ag(I) clusters and the deprotonated cage **1**^**3–**^ can be estimated in the same manner as for neutral capsules (Figure 5). The high values of the interaction energies between the Ag(I) clusters and charged cages coupled with relatively high bond index values between Ag^+^ ions and N_pyrrole_ atoms (with Wiberg bond indices above 0.4) suggest that it is a result of both electrostatic interactions and covalent bonding between these moieties (see Table S4). As discussed previously, we also designed a simplified representation of **[Ag***_**n**_*⊂**1]**^***n*-3+**^ with a reduced number of atoms, composed of 2,5-dimethylpyrrole and trimethylamine moieties. As in the case of **Ag**_**2**_**Cl**_**2**_⊂**1-H**_**3**_ and **[Ag**_**2**_**F**⊂**1-H**_**3**_**]**^**+**^, for **Ag**_**3**_⊂**1**, we obtained a similar geometry with the RMSD between these atoms in the full-atom and reduced system equal to 1.14 Å, see Figure 5. We also designed a system with an additional trimethylamine to produce an identical geometry for the Ag cluster but with stronger interactions between molecules and ions, leading to DFT interaction energies of −1117.7 kcal/mol for the complex in Figure 5G and −1127.4 kcal/mol for the complex in Figure 5H. Interestingly, in the case of Ag_4_^4+^ and Ag_5_^5+^ clusters, such reduced models of cage **1** lead to entirely different geometries for the silver clusters (see the Supporting Information). This structural variability resembles the stochastic dynamic behavior for the formation of higher-order Ag_n_^n+^ clusters observed during the experimental investigations,^29^ which is analyzed at the end of this study.

**Figure 6:**
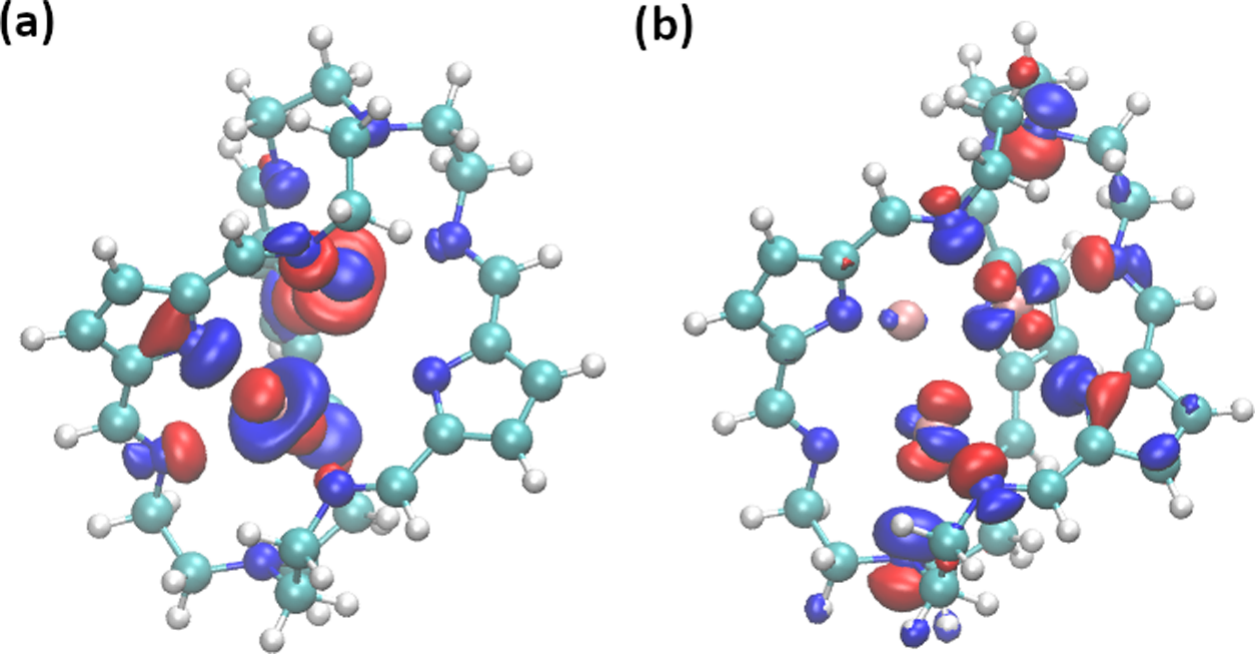
Selected frontier orbitals of the studied systems: (a) HOMO–3 of **[Ag**_**2**_⊂**1]**^**–**^ and (b) HOMO–3 of **Ag**_**3**_⊂**1**.

The analysis of the frontier orbitals and bond indices (see Figure 6 and Tables 3, S5, and S6) provides interesting data regarding possible argentophilic interactions between the Ag atoms. In all studied **[Ag***_**n**_*⊂**1]**^***n*-3+**^ systems (apart from **[Ag**⊂**1]**^**2–**^ with only a single Ag atom), we can identify a relatively strong Ag–Ag argentophilic interaction between these atoms, with a WBI of at least 0.18, with the highest value of 0.35 for these two atoms in the **Ag**_**3**_⊂**1** system. For this cage, we can also identify frontier orbitals with relatively significant contributions on the Ag atoms; see Figure 6b. A thorough analysis of bond indices showed that the most important interactions are between Ag clusters and the N pyrrole and N imine fragments along with non-negligible interactions between Ag ions. The WBI obtained in this study can be compared with those of other, similar systems synthesized earlier, including complexes with Ag_2_^2+^ and Ag_3_^3+^ clusters.^77,78^ In both cases, the bond indices are lower than for the systems studied here, with maximum values of 0.23; see Figure S2 and Table S7. However, it is important to note that when using the all-electron WTBS basis set to describe Ag atoms, all calculated bond indices were lower, see Table S5 for the **Ag**_**3**_⊂**1** case. The analysis of the partial charges of Ag and N atoms reveals that the negative charge of the cage is delocalized over the entire molecule and, in particular, on the pyrrole and imine N atoms. We find that the higher the number of Ag atoms inside the cage, the stronger the overall interactions between Ag atoms and the cage. This is clearly visible in the higher partial charges of Ag atoms and lower partial charges of N atoms (mainly pyrrolides) when considering systems with additional Ag atoms.

**Table 3:** Selected Total or Average Wiberg Bond Order Values and Total or Average Partial Charges (in |e|) for **[Ag**_**n**_⊂ **1]**^**n-3+**^

bond	Wiberg index	atom	Mulliken partial charge	natural partial charge
**[Ag**_**1**_⊂**1]**^**2–**^				
Ag–N_pyrrole_	0.44	Ag	0.19	0.58
Ag–N_imine_	0.12	N_pyrrole_	–0.46	–0.53
		N_imine_	–0.33	–0.48
**[Ag**_**2**_⊂**1]**^**–**^				
Ag–Ag	0.30	Ag	0.23	0.56
Ag–N_pyrrole_	0.28	N_pyrrole_	–0.48	–0.51
Ag–N_imine_	0.37	N_imine_	–0.35	–0.52
**Ag**_**3**_⊂**1**				
Ag–Ag	0.28	Ag	0.25	0.59
Ag–N_pyrrole_	0.22	N_pyrrole_	–0.57	–0.59
Ag–N_imine_	0.25	N_imine_	–0.39	–0.53
**[Ag**_**4**_**-A**⊂**1]**^**+**^				
Ag–Ag	0.21	Ag	0.29	0.59
Ag–N_pyrrole_	0.18	N_pyrrole_	–0.59	–0.63
		N_imine_	–0.40	–0.56
**[Ag**_**4**_**-B**⊂**1]**^**+**^				
Ag–Ag	0.20	Ag	0.29	0.59
Ag–N_pyrrole_	0.18	N_pyrrole_	–0.57	–0.62
		N_imine_	–0.41	–0.57
**[Ag**_**5**_⊂**1]**^**2+**^				
Ag–Ag	0.18	Ag	0.32	0.59
Ag–N_pyrrole_	0.17	N_pyrrole_	–0.64	–0.68
		N_imine_	–0.43	–0.57

Interestingly, five silver ions inside the cage constituted an apparent limit during experimental investigations, yet our theoretical calculations for the system with six Ag^+^ ions inside the cage did converge during geometry optimization. The Ag_6_^6+^ cluster in the resulting complex **[Ag**_**6**_⊂**1]**^**3+**^ adopted a square bipyramidal geometry. Furthermore, starting from the optimized geometry of **[Ag**_**6**_⊂**1]**^**3+**^, we manually added another Ag^+^ ion at the centroid of the square bipyramid to form complex **[Ag**_**7**_⊂**1]**^**4+**^ so that the centroid Ag^+^ ion would acquire an octahedral geometry. After the geometry optimization of **[Ag**_**7**_⊂**1]**^**4+**^, the Ag_7_^7+^ cluster rearranged into the Ag_5_^5+^ trigonal bipyramidal configuration with two Ag^+^ ions independently bonded to the same Ag^+^ vertex. These results suggest that higher-order Ag*_n_*^*n*+^ clusters can be indeed encapsulated into pyrrolide cages so that we encourage further experimental investigations (e.g., imine chain modifications).

**Figure 7:**
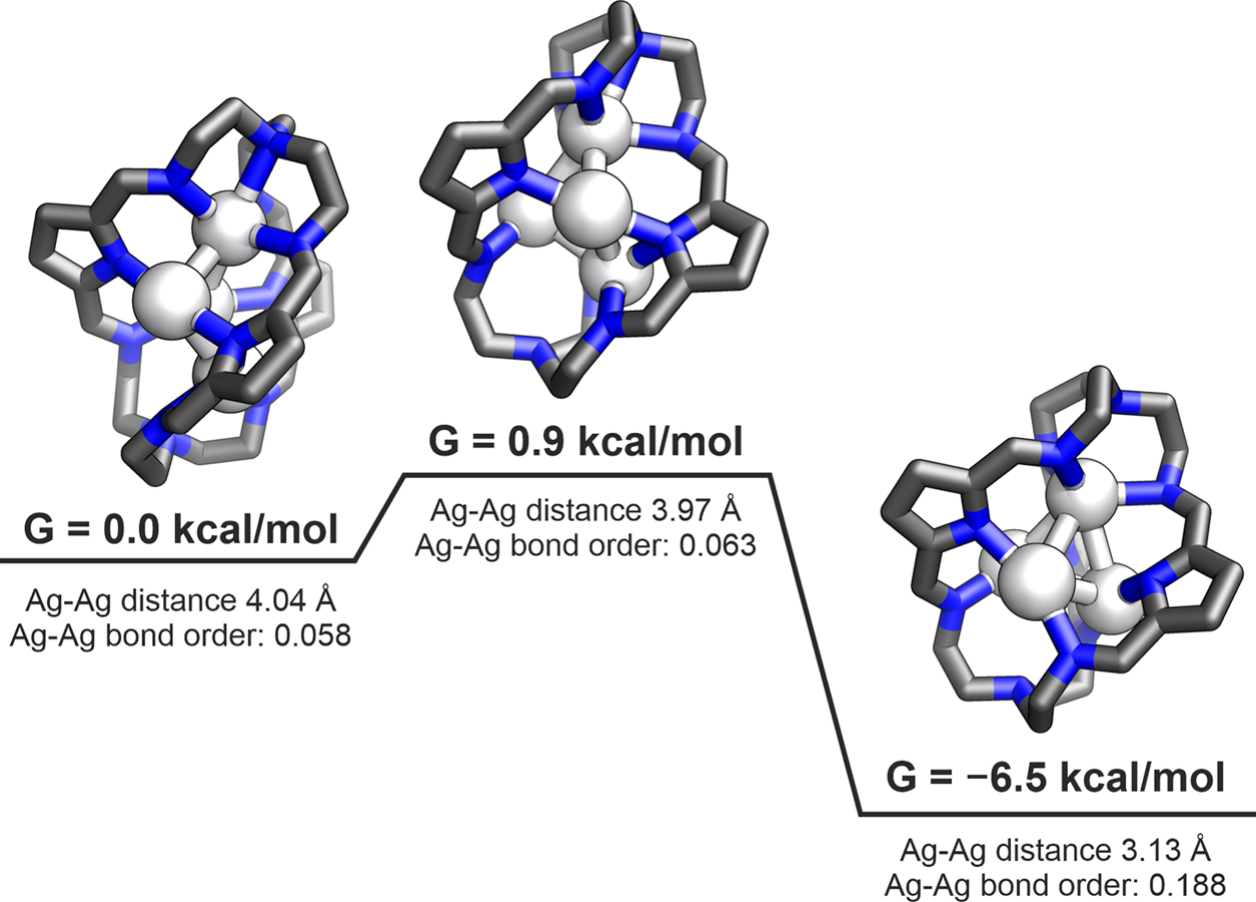
Potential energy surface of the transition from **[Ag**_**4**_**-A**⊂**1]**^**+**^ to **[Ag**_**4**_**-B**⊂**1]**^**+**^.

For the **[Ag**_**4**_⊂**1]**^**+**^ system, two distinct structures/conformations with different geometries of Ag clusters were found by X-ray investigations. Here, we calculated their relative energies/Gibbs free energies and estimated the energy barrier of the transition state connecting these two conformations (see Figure 7). The very small activation barrier of **[Ag**_**4**_**-A**⊂**1]**^**+**^ (Δ*G* = 0.9 kcal/mol) and the very early transition state suggest that this system is very dynamic, readily shifting between the two conformers, even at room temperature. This result is validated by a short (5 ns) molecular dynamics (MD) run under experimental conditions (*T* = 350 K, acetonitrile) starting with **[Ag**_**4**_**-B**⊂**1]**^**+**^. During the 5 ns time scale, the system shifts between these conformers more than 50 times (see Figure S3 and the MD animations in the Supporting Information). Furthermore, the entire Ag_4_ cluster also rotates inside the cage, with Ag atoms constantly changing their closest neighbors, interacting with various N atoms of the cage (see Figure S4). In analogous MD runs of other complexes, for example, in **Ag**_**3**_⊂**1**, the Ag clusters are much more stable because their trigonal geometrical configuration remained unaltered (see Figure S5). Furthermore, cluster rotation inside the cage was also observed due to Ag–N bond-length variations evaluated with the closest nitrogen atoms of cage **1** (see Figure S6 and MD animations in the Supporting Information).

**Figure 8:**
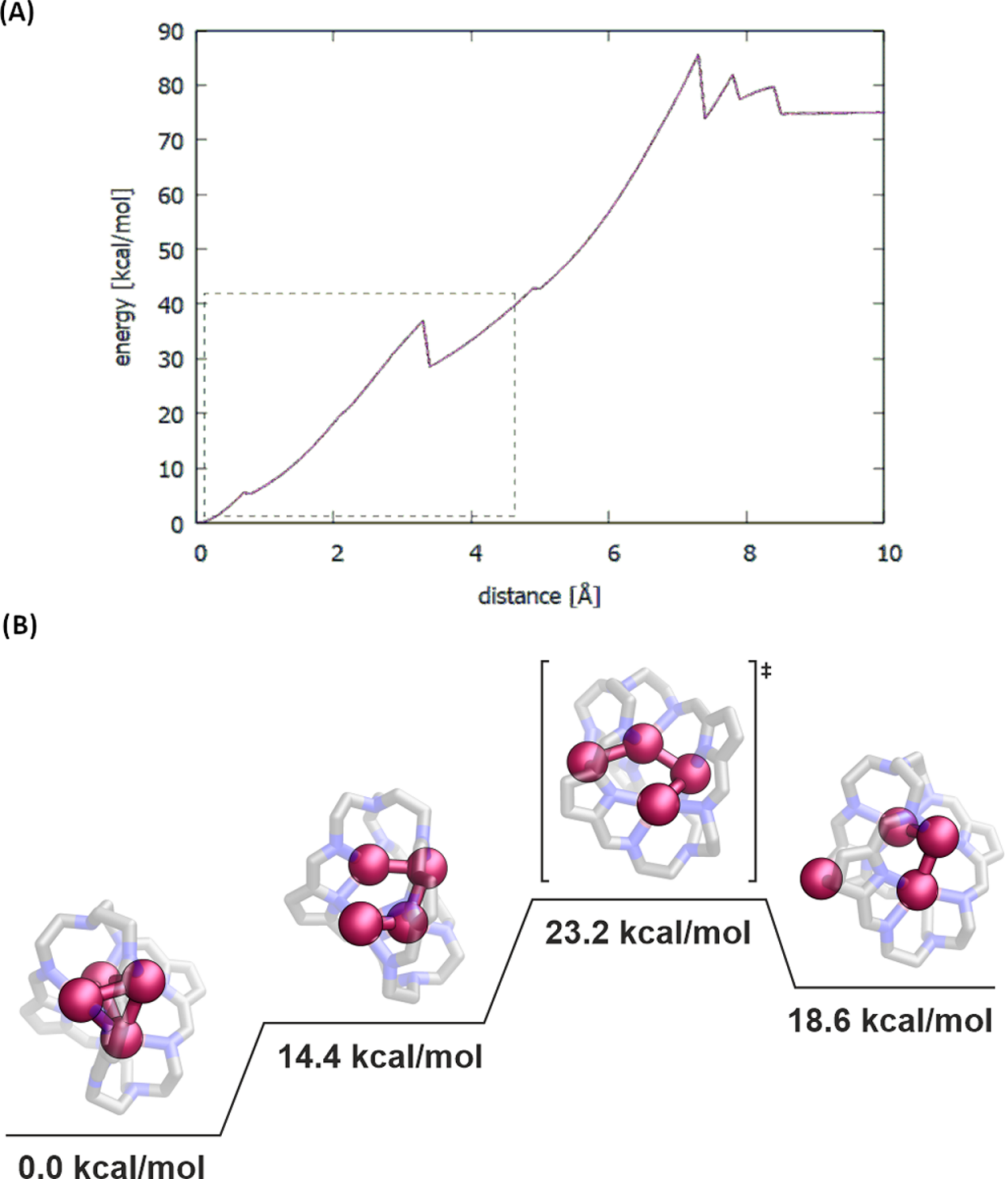
(A) Potential energy surface (PES) of a single Ag^+^ ion exiting the inside of **1**^**3–**^ for **[Ag**_**4**_**-B**⊂**1]**^**+**^ (the distance is relative to the optimal position of the pulled Ag^+^ ion) and (B) part of the PES of a single Ag^+^ ion entering the **Ag**_**3**_⊂**1** to form **[Ag**_**4**_**-B**⊂**1]**^**+**^ calculated at the DFT level of theory.

Finally, we also considered the formation of **[Ag***_**n**_*⊂**1]**^***n*-3+**^ complexes by scanning the potential energy surface (PES) of a single Ag^+^ ion exiting the interior of **1** at the GFN2-xTB level of theory (see Figure S7). Figure 8A shows the PES scan for **[Ag**_**4**_**-B**⊂**1]**^**+**^, suggesting two barriers to the entry of Ag^+^ into **Ag**_**3**_⊂**1** to form **[Ag**_**4**_**-B**⊂**1]**^**+**^, both less than 10 kcal/mol. For this system, we also performed studies at the DFT level of theory, locating a stationary point of Ag^+^ outside of **1** that interacts with two pyrrole moieties (18.6 kcal/mol with respect to the **[Ag**_**4**_**-B**⊂**1]**^**+**^), followed by a transition state of Ag^+^ entering **1** (23.2 kcal/mol), a stationary point with the Ag^+^ ion inside **1** but not in optimal position (14.4 kcal/mol), and finally the fully optimized structure of **[Ag**_**4**_**-B**⊂**1]**^**+**^ (0.0 kcal/mol); see Figure 8B. These results agree with the experimental data, suggesting a relatively easy formation of such complexes and conversion from systems with fewer Ag^+^ ions to systems with more Ag^+^ ions as soon as additional silver(I) is introduced.^29^ In all other cases, the barriers are of similar height, apart from when the Ag^+^ ion enters the empty cage which has an energy barrier of around 20 kcal/mol. In most cases, there are multiple local minima, corresponding to geometries where Ag^+^ ions have not entered the cavity of **1**, but interact with selected parts of **1**. Also, Ag^+^ ions exiting/entering **1** are “sandwiched” by two pyrrole rings, which seems to be energetically favorable due to cation–π noncovalent interactions. The animations visualizing Ag^+^ ions paths entering or exiting the cage are presented in the Supporting Information.

### Pyrrolide Cages Incorporating Mixed-Valence Silver Clusters

The effect of the hypothetical encapsulated clusters consisting of metallic silver and silver(I) ions on structural variability was also investigated (a detailed analysis is reported in the Supporting Information). For cages that incorporate silver(I) clusters **[Ag***_**n**_*⊂**1]**^***n*-3+**^, we gradually replaced Ag^+^ by Ag^0^ in the Ag*_n_*^*n*+^ cluster resulting in a series of mixed-valence ^*m*^[**Ag***_**n**_*⊂**1**]^*q*^ complexes with *n* = 3–5. The multiplicity *m* of these systems was varied from singlet to triplet or from doublet to quartet as appropriate, and the charge *q* ranged from (*n* – 3) to −3. We also included the effect of environment polarization due to solvent, hence geometries of these systems were optimized in the gas phase and in dichloromethane (dielectric constant ε = 8.93), 1-propanol (ε = 20.52), and acetonitrile (ε = 35.69). Our findings revealed that the phase (gas or solution) did not alter the geometry of the cluster inside the cage **1**^**3–**^. On the other hand, access to alternative geometrical configurations was attained through the formation of mixed-valence clusters, rather than being induced by environment polarization. It is crucial to emphasize that all DFT geometry optimizations for mixed-valence ^*m*^[**Ag***_**n**_*⊂**1**]^*q*^ structures started from the optimized silver(I) complexes **[Ag***_**n**_*⊂**1]**^***n*-3+**^; this is particularly important because the formation of these complexes from the respective reactants was indeed altered by the choice of solvent and relative solubility of individual constituents. Our results indicate that once a complex is synthesized, subsequent alterations in the environment polarization are not expected to modify the respective geometrical conformation. On the other hand, the incorporation of metallic silver reinforces the experimental conclusion that the reaction results depend on a series of intricate processes such as ion exchange, (de)complexation, (de)protonation, and anion-binding events through the cage **1**^**3–**^ (Ag^0^ evidently would limit some of these processes).^29^ For example, in complexes ^*m*^[**Ag**_**5**_⊂**1**]^*q*^ with *m* = 1–4 and *q* = +2 and +1, the Ag_5_ trigonal bipyramid remained unaltered, but the substitution of two Ag^+^ by two Ag^0^ leading to structure ^1^[**Ag**_**5**_⊂**1**]^0^ resulted in a geometrical rearrangement characterized as Ag_5_ square pyramid. Furthermore, we observed a similar trend in the anionic structures ^2^[**Ag**_**5**_⊂**1**]^1–^, ^1^[**Ag**_**5**_⊂**1**]^2–^, and ^3^[**Ag**_**5**_⊂**1**]^2–^, which were characterized as Ag_5_ distorted trigonal bipyramid, i.e., an intermediate structure between the trigonal bipyramid and square pyramid (see Tables S8–S10 and Figure S8).

The mixed-valence DFT approach to ^*m*^[**Ag**_**4**_⊂**1**]^*q*^ resulted in the same conclusions as attained from our MD simulations detailed in the previous subsection. (i) The Ag_4_ cluster can rotate inside cage **1**^**3**–^, (ii) the Ag_4_ trigonal pyramid in ^*m*^[**Ag**_**4**_**-B**⊂**1**]^*q*^ is thermodynamically more stable than the Ag_4_ rhomboid in ^*m*^[**Ag**_**4**_**-A**⊂**1**]^*q*^, and (iii) the Ag_4_ cluster exhibited several trigonal pyramidal conformers. We observed two additional geometrical configurations in the case of *q* = −3: a planar rhomboid ^1^[**Ag**_**4**_**A**⊂**1**]^3–^ and a trigonal pyramid ^3^[**Ag**_**4**_**B**⊂**1**]^3–^ characterized by a high localization of the apex (see Tables S11–S14 and Figure S9). However, these structures are thermodynamically destabilized or spin-contaminated, respectively. Furthermore, in line with the MD results described earlier, the mixed-valence Ag_3_ trigonal cluster in ^*m*^[**Ag**_**3**_⊂**1**]^*q*^ exhibited a reduced degree of structural variability (see Table S15 and Figure S10); essentially, the Ag_3_ cluster can only rotate inside the cage **1**^**3**–^.

**Figure 9:**
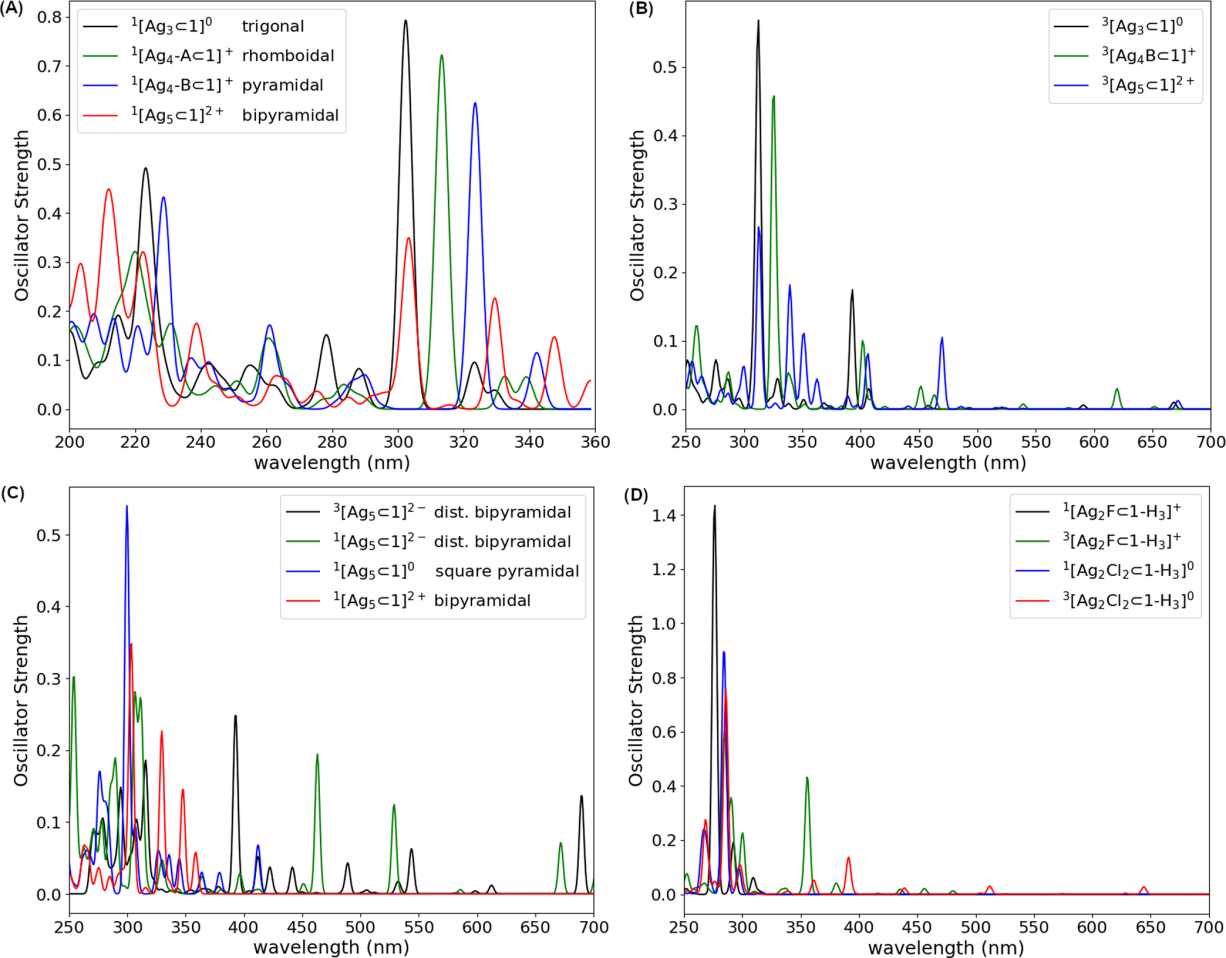
(A) Singlet-to-singlet and (B) triplet-to-triplet time-dependent DFT spectra in the gas phase calculated for pyrrolide cages incorporating silver(I) clusters. Calculated spectra for cryptates encapsulating (C) mixed-valence silver clusters and (D) silver(I) halide salts.

Finally, we calculated gas-phase time-dependent DFT electronic excitations in silver(I) and mixed-valence ^*m*^[**Ag**_**n**_⊂**1**]^*q*^ complexes to determine structural variability (spectra in solution and methodological aspects are detailed in the Supporting Information, see Figures S11–S20). Interestingly, UV–vis spectra may be used as a diagnostic probe to distinguish between planar and pyramidal conformations so that the main absorption bands, specifically in the 300–350 nm region, are blue-shifted and more intense for planar geometrical configurations (see Figure 9A). The silver(I) **[Ag***_**n**_*⊂**1]**^***n*-3+**^ systems exhibited weak absorptions in the visible region calculated via triplet-to-triplet transitions (Figure 9B) so that the nontransparent physical aspect of these compounds can be attributed to high-spin electronic transitions. Furthermore, our results revealed absorption bands in the visible region for mixed-valence ^*m*^[**Ag***_**n**_*⊂**1**]^*q*^ complexes (Figure 9C), which are absent in the singlet-to-singlet spectra calculated for the cage that incorporates silver(I) **[Ag***_**n**_*⊂**1]**^***n*-3+**^. The spectra calculated for the cryptates incorporating silver(I) halide salts **[Ag**_**2**_**F**⊂**1-H**_**3**_**]**^**+**^ and **Ag**_**2**_**Cl**_**2**_⊂**1-H**_**3**_ exhibited a similar trend to the silver(I) **[Ag***_**n**_*⊂**1]**^***n*-3+**^ structures: (i) main UV-light abortions due to singlet-to-singlet transitions and (ii) weak visible-light absorptions as a result of triplet-to-triple transitions. Particularly, weak absorptions of purple-color light at ca. 450 nm (see the green line in Figure 9D) may be attributed to the yellow color of compound **[Ag**_**2**_**F**⊂**1-H**_**3**_**]**^**+**^ determined experimentally. On the other hand, the pale, pink-colored physical aspect of **Ag**_**2**_**Cl**_**2**_⊂**1-H**_**3**_ could not be unambiguously assigned by means of our calculations because the weak absorptions were localized at several points of the visible region.

Finally, we also analyzed the bond indices, similarly to the case of nonmixed valence clusters, to search for possible argentophilic interactions. In most cases, the calculated bond indices for mixed-valence clusters are similar to those obtained previously for the original systems (see Table S16) with most WBI values between Ag atoms/ions below 0.4. The only exception to this rule is the **[Ag**_**3**_⊂**1]**^**3–**^ system with all three Ag atoms bearing a formal zero charge, where the WBI values are between 0.62 and 0.66, indicating a largely covalent character of interactions between the Ag atoms. Interestingly, in no other system, including **[Ag**_**4**_⊂**1]**^**3–**^ and **[Ag**_**5**_⊂**1]**^**3–**^, we were able to observe WBI indices of 0.5 or more. The likely explanation of this result is the fact that in the **[Ag**_**3**_⊂**1]**^**3–**^ system Ag atoms are closer to each other (with an average distance of 2.7 Å) than in **[Ag**_**4**_⊂**1]**^**x**^ (average distance of above 3 Å) or **[Ag**_**5**_⊂**1]**^**x**^ (>2.8 Å).

## Conclusions

In this study, we report the geometric and electronic properties (both static and dynamic) of the recently synthesized pyrrole cages encapsulating ion pairs and silver(I) clusters. We show that for neutral pyrrole cages, the Gibbs free energies of formation provide good measures for predicting the ratio of bound ions, and we predict that Br^–^ and I^–^ ions will preferably form **Ag**_**2**_**X**_**2**_⊂**1-H**_**3**_-type cryptands. For the charged pyrrole cages incorporating silver(I) clusters, we identified moderately strong argentophilic interactions between silver(I) ions based on the calculated bond indices and molecular orbitals analysis. The dynamics results for **[Ag**_**4**_⊂**1]**^**+**^ cryptate reveals that the two minimum-geometry conformations differ by only 6.5 kcal/mol, with an energy barrier of less than 1 kcal/mol, suggesting a very flexible structure, which is further supported by the molecular dynamics results. This observation is associated with a behavior previously termed coordination plasticity of cryptates. Finally, we estimated the energy barriers of the formation of **[Ag***_**n**_*⊂**1]**^**n-3+**^ (*n* = 1–5) cryptates, showing relatively low energy barriers of formation for all of them, consistent with experimental data. Our investigations of the reduced cage models reveal that both the neutral and deprotonated forms of the cage are very flexible and, to a large extent, can adjust their conformations depending on the guest molecule to maximize host–guest interactions. Furthermore, the geometrical conformation of silver(I) clusters inside the cage can be altered by means of the replacement of Ag^+^ ions with metallic silver.

Finally, we suggest a diagnostic probe based on calculated UV–vis spectra to characterize the geometrical configurations of silver clusters inside the cage. In this context, main bands in planar conformations are expected to absorb more intensely at shorter wavenumbers, and triplet-to-triple electronic transitions or mixed-valence silver clusters are predicted to absorb in the visible region.

## Supporting Information Available

Bond orders, partial charges, interaction energies, DFT characterization, and TDDFT spectra for studied systems. Graphical analysis of MD runs and potential energy surfaces of Ag^+^ ions exiting the cavity of **1** (PDF)
Cartesian coordinates of all studied systems (XYZ)
Animation of MD runs and Ag^+^ ions exiting the cavity of **1** (MP4)
Animation of MD runs and Ag^+^ ions exiting the cavity of **1** (MP4)
Animation of MD runs and Ag^+^ ions exiting the cavity of **1** (MP4)
Animation of MD runs and Ag^+^ ions exiting the cavity of **1** (MP4)
Animation of MD runs and Ag^+^ ions exiting the cavity of **1** (MP4)
Animation of MD runs and Ag^+^ ions exiting the cavity of **1** (MP4)

## Supplementary Material

jp4c01464_si_001.pdf

jp4c01464_si_002.xyz
