# Supplementary material for: Argentophilic Interactions, Flexibility, and Dynamics of Pyrrole Cages Encapsulating Silver(I) Clusters
Source: J Phys Chem A. 2024 Apr 23;128(17):3339–50. doi: 10.1021/acs.jpca.4c01464 (PMC11077489; doi:10.1021/acs.jpca.4c01464)
Supplement: Supplementary file 1 — jp4c01464_si_001.pdf [file jp4c01464_si_001.pdf]

Argentophilic Interactions, Flexibility and Dynamics of Pyrrole Cages Encapsulating Silver(I) Clusters

Bartosz Trzaskowski,† Juan Pablo Martínez,† Aleksandra Sarwa,ǂ Bartosz Szyszko,*ǂ and William A. Goddard III§*

† Centre of New Technologies, University of Warsaw, 2C Banacha St., 02-097 Warszawa, Poland.

ǂ Faculty of Chemistry, University of Wrocław, 14 F. Joliot-Curie St., 50-387 Wrocław, Poland

§ Materials and Process Simulation Center, California Institute of Technology, Pasadena, California 91106, United States

Table S1. Differences in relative energies between the lowest-energy conformations of **1-H$_3$** and **1^{3-}**.

conformation	relative energy (kcal/mol)
**1-H$_3$**	
conformation 1	0.0
conformation 2	0.1
conformation 3	0.2
conformation 4	0.6

conformation 5	1.8
**1^{3-}**	
conformation 1	0.0
conformation 2	7.1
conformation 3	7.2
conformation 4	9.9
conformation 5	10.8

Table S2. Selected Wiberg, Mayer and fuzzy bond order values for **Ag_2Cl_2⊂1-H_3** and [**Ag_2F⊂1-H_3**]$^+$.

bond	Wiberg index	Mayer index	Fuzzy index
**Ag_2Cl_2⊂1-H_3**			
Ag(1)-Ag(2)	0.10	<0.05	0.11
Ag(1)-Cl(1)	0.54	0.36	0.75
Ag(1)-Cl(2)	0.56	0.36	0.78
Ag(2)-Cl(1)	0.54	0.36	0.75
Ag(2)-Cl(2)	0.56	0.36	0.78
Ag(1)-N_{amine}(1)	0.27	0.19	0.55
Ag(1)-N_{imine}(1)	0.30	0.22	0.57
Ag(1)-N_{imine}(2)	0.31	0.22	0.57
Ag(1)-N_{imine}(3)	0.13	0.09	0.27
Ag(2)-N_{amine}(2)	0.27	0.19	0.55
Ag(2)-N_{imine}(4)	0.31	0.22	0.57
Ag(2)-N_{imine}(5)	0.30	0.22	0.57
Ag(2)-N_{imine}(6)	0.13	0.09	0.27

**[$Ag_2F \subset$1-H_3]$^+$**			
Ag(1)-Ag(2)	<0.05	<0.05	<0.05
Ag(1)-F(1)	0.45	0.28	0.77
Ag(2)-F(1)	0.45	0.28	0.77
Ag(1)-N_{amine}(1)	0.27	0.17	0.55
Ag(1)-N_{imine}(1)	0.45	0.30	0.77
Ag(1)-N_{imine}(2)	0.45	0.30	0.77
Ag(1)-N_{imine}(3)	0.45	0.30	0.77
Ag(2)-N_{amine}(2)	0.27	0.17	0.55
Ag(2)-N_{imine}(4)	0.45	0.30	0.77
Ag(2)-N_{imine}(5)	0.45	0.30	0.77
Ag(2)-N_{imine}(6)	0.45	0.30	0.77

Table S3. Selected partial charges (in |e|) for **$Ag_2Cl_2 \subset$ 1-H_3** and [**$Ag_2F \subset$ 1-H_3]$^+$**.

atom	Mulliken	NBO
**$Ag_2Cl_2 \subset$1-H_3**		
Ag(1)	0.14	0.51
Ag(2)	0.14	0.51
Cl(1)	-0.49	-0.71
Cl(2)	-0.49	-0.71
N_{amine}(1)	-0.43	-0.55
N_{imine}(1)	-0.33	-0.48
N_{imine}(2)	-0.34	-0.49
N_{imine}(3)	-0.33	-0.45
N_{amine}(2)	-0.43	-0.55

N_{imine}(4)	-0.34	-0.49
N_{imine}(5)	-0.33	-0.48
N_{imine}(6)	-0.33	-0.45
**[Ag_2F⊂1-H_3]$^+$**		
Ag(1)	0.20	0.57
Ag(2)	0.20	0.57
F	-0.46	-0.68
N_{amine}(1)	-0.44	-0.56
N_{imine}(1)	-0.38	-0.52
N_{imine}(2)	-0.38	-0.52
N_{imine}(3)	-0.38	-0.52
N_{amine}(2)	-0.44	-0.56
N_{imine}(4)	-0.38	-0.52
N_{imine}(5)	-0.38	-0.52
N_{imine}(6)	-0.38	-0.52

Table S4. Interaction energies between the Ag_n^{n+} and $\mathbf{1}^{3-}$.

system	DFT interaction energy (kcal/mol)	SAPT0 interaction energy (kcal/mol)
**[Ag_1⊂1]$^{2-}$**	-378.8	-365.7
**[Ag_2⊂1]$^{-}$**	-741.2	-712.3
**Ag_3⊂1**	-1132.5	-1096.8
**[Ag_4-A⊂1]$^{+}$**	-1533.9	-1493.8
**[Ag_4-B⊂1]$^{+}$**	-1544.5	-1503.5
**[Ag_5⊂1]$^{2+}$**	-1934.7	-1889.3

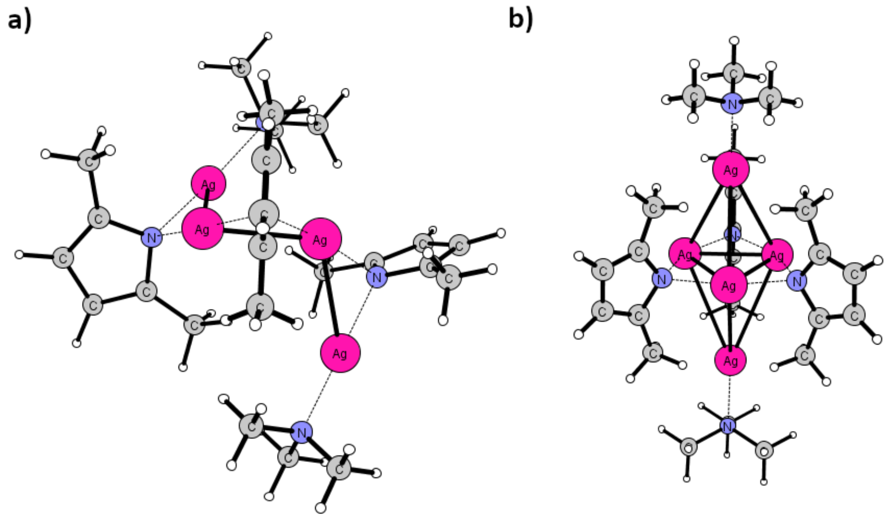

Figure S1. DFT-optimized structures of reduced complex containing three pyrrole and two trimethylamine moieties interacting with a) Ag_4 cluster, and b) Ag_5 cluster.

Table S5. Selected Wiberg, Mayer and fuzzy bond order values for **[Ag$_n$ ⊂ 1]$^{n-3}$**. Values in parentheses correspond to calculations performed in the 6-31G**//WTBS basis set.

bond	Wiberg index	Mayer index	Fuzzy index
**[Ag$_1$⊂1]$^{2-}$**			
Ag(1)-N$_{pyrrole}$(1)	0.46	0.32	0.77
Ag(1)-N$_{pyrrole}$(2)	0.45	0.31	0.75
Ag(1)-N$_{pyrrole}$(3)	0.40	0.28	0.68
**[Ag$_2$⊂1]$^{-}$**			
Ag(1)-Ag(2)	0.30	0.15	0.51
Ag(1)-N$_{pyrrole}$(1)	0.43	0.35	0.72
Ag(1)-N$_{pyrrole}$(2)	0.07	<0.05	0.08
Ag(1)-N$_{pyrrole}$(3)	0.21	0.17	0.40
Ag(1)-N$_{tert}$(1)	0.06	<0.05	0.16

Ag(1)-N_{imine}(1)	0.31	0.21	0.59
Ag(1)-N_{imine}(2)	0.37	0.26	0.69
Ag(1)-N_{imine}(3)	0.41	0.27	0.74
Ag(2)-$N_{pyrrole}$(1)	0.09	0.15	0.12
Ag(2)-$N_{pyrrole}$(2)	0.44	0.35	0.75
Ag(2)-$N_{pyrrole}$(3)	0.09	<0.05	0.16
Ag(2)-N_{tert}(2)	0.08	<0.05	0.22
Ag(2)-N_{imine}(4)	0.34	0.24	0.64
Ag(2)-N_{imine}(5)	0.38	0.27	0.70
Ag(2)-N_{imine}(6)	0.42	0.28	0.75
**Ag_3⊂1**			
Ag(1)-Ag(2)	0.19 (<0.05)	0.07 (<0.05)	0.28 (0.17)
Ag(1)-Ag(3)	0.35 (0.05)	0.13 (<0.05)	0.53 (0.39)
Ag(2)-Ag(3)	0.29 (<0.05)	0.08 (<0.05)	0.42 (0.25)
Ag(1)-$N_{pyrrole}$(1)	<0.05 (<0.05)	<0.05 (<0.05)	<0.05 (<0.05)
Ag(1)-$N_{pyrrole}$(2)	0.10 (<0.05)	<0.05 (<0.05)	0.14 (0.17)
Ag(1)-$N_{pyrrole}$(3)	0.08 (<0.05)	<0.05 (<0.05)	0.10 (0.10)
Ag(1)-N_{tert}(1)	0.22 (0.06)	0.14 (<0.05)	0.49 (0.49)
Ag(1)-N_{imine}(1)	0.38 (0.15)	0.29 (0.16)	0.68 (0.68)
Ag(1)-N_{imine}(2)	0.41 (0.11)	0.34 (0.10)	0.74 (0.49)
Ag(1)-N_{imine}(3)	0.49 (0.20)	0.36 (0.20)	0.84 (0.80)
Ag(2)-$N_{pyrrole}$(1)	0.08 (<0.05)	<0.05 (<0.05)	0.11 (0.07)
Ag(2)-$N_{pyrrole}$(2)	0.05 (<0.05)	<0.05 (<0.05)	<0.05 (<0.05)
Ag(2)-$N_{pyrrole}$(3)	0.52 (0.24)	0.42 (0.25)	0.86 (0.82)
Ag(2)-N_{tert}(2)	<0.05 (<0.05)	<0.05 (<0.05)	0.07 (0.06)
Ag(2)-N_{imine}(4)	<0.05 (<0.05)	<0.05 (<0.05)	<0.05 (<0.05)

Ag(2)-N_{imine}(5)	0.48 (0.22)	0.38 (0.23)	0.82 (0.81)
Ag(2)-N_{imine}(6)	0.40 (0.12)	0.29 (0.10)	0.71 (0.67)
Ag(3)-$N_{pyrrole}$(1)	0.62 (0.33)	0.54 (0.33)	1.00 (0.96)
Ag(3)-$N_{pyrrole}$(2)	0.57 (0.26)	0.52 (0.26)	0.93 (0.85)
Ag(3)-$N_{pyrrole}$(3)	<0.05 (<0.05)	<0.05 (<0.05)	<0.05 (<0.05)
Ag(3)-N_{tert}(1)	<0.05 (<0.05)	<0.05 (<0.05)	<0.05 (<0.05)
Ag(3)-N_{imine}(1)	0.08 (<0.05)	<0.05 (<0.05)	0.11 (0.09)
Ag(3)-N_{imine}(2)	0.05 (<0.05)	<0.05 (<0.05)	0.05 (<0.05)
Ag(3)-N_{imine}(3)	<0.05 (<0.05)	<0.05 (<0.05)	<0.05 (<0.05)
**[Ag_4-A⊂1]$^+$**			
Ag(1)-Ag(2)	0.30	0.13	0.48
Ag(1)-Ag(3)	0.21	0.09	0.32
Ag(1)-Ag(4)	0.06	<0.05	<0.05
Ag(2)-Ag(3)	0.14	<0.05	0.14
Ag(2)-Ag(4)	0.26	0.11	0.41
Ag(3)-Ag(4)	0.27	0.09	0.39
Ag(1)-$N_{pyrrole}$(1)	<0.05	<0.05	<0.05
Ag(1)-$N_{pyrrole}$(2)	0.10	<0.05	0.13
Ag(1)-$N_{pyrrole}$(3)	0.07	<0.05	0.08
Ag(2)-$N_{pyrrole}$(1)	0.50	0.43	0.81
Ag(2)-$N_{pyrrole}$(2)	0.54	0.48	0.89
Ag(2)-$N_{pyrrole}$(3)	<0.05	<0.05	<0.05
Ag(3)-$N_{pyrrole}$(1)	<0.05	<0.05	<0.05
Ag(3)-$N_{pyrrole}$(2)	0.07	<0.05	0.11
Ag(3)-$N_{pyrrole}$(3)	0.57	0.46	0.95
Ag(4)-$N_{pyrrole}$(1)	0.29	0.21	0.47

Ag(4)-$N_{pyrrole}$(2)	<0.05	<0.05	<0.05
Ag(4)-$N_{pyrrole}$(3)	0.05	<0.05	0.05
**[Ag_4-B⊂1]$^+$**			
Ag(1)-Ag(2)	0.19	0.07	0.26
Ag(1)-Ag(3)	0.19	0.07	0.27
Ag(1)-Ag(4)	0.19	0.07	0.26
Ag(2)-Ag(3)	0.21	0.06	0.28
Ag(2)-Ag(4)	0.21	0.06	0.28
Ag(3)-Ag(4)	0.21	0.06	0.28
Ag(1)-$N_{pyrrole}$(1)	0.08	<0.05	0.10
Ag(1)-$N_{pyrrole}$(2)	0.08	<0.05	0.10
Ag(1)-$N_{pyrrole}$(3)	0.08	<0.05	0.11
Ag(2)-$N_{pyrrole}$(1)	0.54	0.49	0.88
Ag(2)-$N_{pyrrole}$(2)	<0.05	<0.05	<0.05
Ag(2)-$N_{pyrrole}$(3)	0.11	<0.05	0.16
Ag(3)-$N_{pyrrole}$(1)	<0.05	<0.05	<0.05
Ag(3)-$N_{pyrrole}$(2)	0.11	<0.05	0.16
Ag(3)-$N_{pyrrole}$(3)	0.54	0.49	0.88
Ag(4)-$N_{pyrrole}$(1)	0.11	<0.05	0.16
Ag(4)-$N_{pyrrole}$(2)	0.54	0.49	0.88
Ag(4)-$N_{pyrrole}$(3)	<0.05	<0.05	<0.05
**[Ag_5⊂1]$^{2+}$**			
Ag(1)-Ag(2)	0.26	0.11	0.40
Ag(1)-Ag(3)	<0.05	<0.05	<0.05
Ag(1)-Ag(4)	0.19	0.08	0.27
Ag(1)-Ag(5)	0.25	0.09	0.37

Ag(2)-Ag(3)	0.26	0.11	0.40
Ag(2)-Ag(4)	0.12	<0.05	0.12
Ag(2)-Ag(5)	0.12	<0.05	0.12
Ag(3)-Ag(4)	0.25	0.09	0.37
Ag(3)-Ag(5)	0.19	0.08	0.27
Ag(4)-Ag(5)	0.18	0.05	0.24
Ag(1)-$N_{pyrrole}$(1)	0.18	0.12	0.27
Ag(1)-$N_{pyrrole}$(2)	<0.05	<0.05	<0.05
Ag(1)-$N_{pyrrole}$(3)	<0.05	<0.05	<0.05
Ag(2)-$N_{pyrrole}$(1)	0.53	0.50	0.87
Ag(2)-$N_{pyrrole}$(2)	<0.05	<0.05	<0.05
Ag(2)-$N_{pyrrole}$(3)	0.53	0.50	0.87
Ag(3)-$N_{pyrrole}$(1)	<0.05	<0.05	<0.05
Ag(3)-$N_{pyrrole}$(2)	<0.05	<0.05	<0.05
Ag(3)-$N_{pyrrole}$(3)	0.18	0.12	0.27
Ag(4)-$N_{pyrrole}$(1)	0.07	<0.05	0.09
Ag(4)-$N_{pyrrole}$(2)	0.47	0.41	0.75
Ag(4)-$N_{pyrrole}$(3)	<0.05	<0.05	<0.05
Ag(5)-$N_{pyrrole}$(1)	<0.05	<0.05	<0.05
Ag(5)-$N_{pyrrole}$(2)	0.47	0.41	0.75
Ag(5)-$N_{pyrrole}$(3)	0.07	<0.05	0.09

Table S6. Selected partial charges (in |e|) for **$[Ag_n\subset 1]^{n-3}$**.

Atom	Mulliken	NBO
**$[Ag_1\subset 1]^{2-}$**		
Ag	0.19	0.58
$N_{pyrrole}(1)$	-0.48	-0.54
$N_{pyrrole}(2)$	-0.46	-0.54
$N_{pyrrole}(3)$	-0.46	-0.52
$N_{tert}(1)$	-0.37	-0.53
$N_{imine}(1)$	-0.44	-0.55
$N_{imine}(2)$	-0.30	-0.44
$N_{imine}(3)$	-0.31	-0.45
$N_{tert}(2)$	-0.36	-0.52
$N_{imine}(4)$	-0.30	-0.46
$N_{imine}(5)$	-0.31	-0.48
$N_{imine}(6)$	-0.31	-0.48
**$[Ag_2\subset 1]^{-}$**		
Ag(1)	0.22	0.56
Ag(2)	0.24	0.56
$N_{pyrrole}(1)$	-0.49	-0.53
$N_{pyrrole}(2)$	-0.49	-0.53
$N_{pyrrole}(3)$	-0.46	-0.46
$N_{tert}(1)$	-0.37	-0.52
$N_{imine}(1)$	-0.33	-0.50
$N_{imine}(2)$	-0.34	-0.50
$N_{imine}(3)$	-0.37	-0.54
$N_{tert}(2)$	-0.37	-0.53

$N_{imine}(4)$	-0.34	-0.52
$N_{imine}(5)$	-0.35	-0.51
$N_{imine}(6)$	-0.37	-0.54
**$Ag_3 \subset 1$**		
Ag(1)	0.27	0.49
Ag(2)	0.31	0.60
Ag(3)	0.18	0.59
$N_{pyrrole}(1)$	-0.60	-0.60
$N_{pyrrole}(2)$	-0.57	-0.59
$N_{pyrrole}(3)$	-0.55	-0.58
$N_{tert}(1)$	-0.42	-0.55
$N_{imine}(1)$	-0.36	-0.52
$N_{imine}(2)$	-0.39	-0.55
$N_{imine}(3)$	-0.40	-0.57
$N_{tert}(2)$	-0.40	-0.53
$N_{imine}(4)$	-0.39	-0.47
$N_{imine}(5)$	-0.40	-0.56
$N_{imine}(6)$	-0.38	-0.53
**$[Ag_4\text{-}A \subset 1]^+$**		
Ag(1)	0.27	0.49
Ag(2)	0.23	0.63
Ag(3)	0.30	0.63
Ag(4)	0.36	0.60
$N_{pyrrole}(1)$	-0.62	-0.68
$N_{pyrrole}(2)$	-0.58	-0.61
$N_{pyrrole}(3)$	-0.58	-0.60

N_{tert}(1)	-0.43	-0.56
N_{imine}(1)	-0.37	-0.53
N_{imine}(2)	-0.37	-0.52
N_{imine}(3)	-0.40	-0.55
N_{tert}(2)	-0.40	-0.53
N_{imine}(4)	-0.39	-0.55
N_{imine}(5)	-0.44	-0.59
N_{imine}(6)	-0.41	-0.59
**[Ag_4-B⊂1]$^{+}$**		
Ag(1)	0.26	0.48
Ag(2)	0.31	0.62
Ag(3)	0.30	0.62
Ag(4)	0.30	0.62
$N_{pyrrole}$(1)	-0.57	-0.62
$N_{pyrrole}$(2)	-0.57	-0.62
$N_{pyrrole}$(3)	-0.57	-0.62
N_{tert}(1)	-0.41	-0.55
N_{imine}(1)	-0.38	-0.53
N_{imine}(2)	-0.38	-0.53
N_{imine}(3)	-0.38	-0.53
N_{tert}(2)	-0.40	-0.52
N_{imine}(4)	-0.43	-0.60
N_{imine}(5)	-0.43	-0.60
N_{imine}(6)	-0.43	-0.60
**[Ag_5⊂1]$^{2+}$**		
Ag(1)	0.31	0.49

Ag(2)	0.24	0.64
Ag(3)	0.31	0.49
Ag(4)	0.36	0.67
Ag(5)	0.36	0.67
$N_{pyrrole}(1)$	-0.60	-0.65
$N_{pyrrole}(2)$	-0.71	-0.74
$N_{pyrrole}(3)$	-0.60	-0.65
$N_{tert}(1)$	-0.43	-0.56
$N_{imine}(1)$	-0.41	-0.56
$N_{imine}(2)$	-0.47	-0.61
$N_{imine}(3)$	-0.40	-0.55
$N_{tert}(2)$	-0.43	-0.56
$N_{imine}(4)$	-0.41	-0.56
$N_{imine}(5)$	-0.47	-0.61
$N_{imine}(6)$	-0.40	-0.55

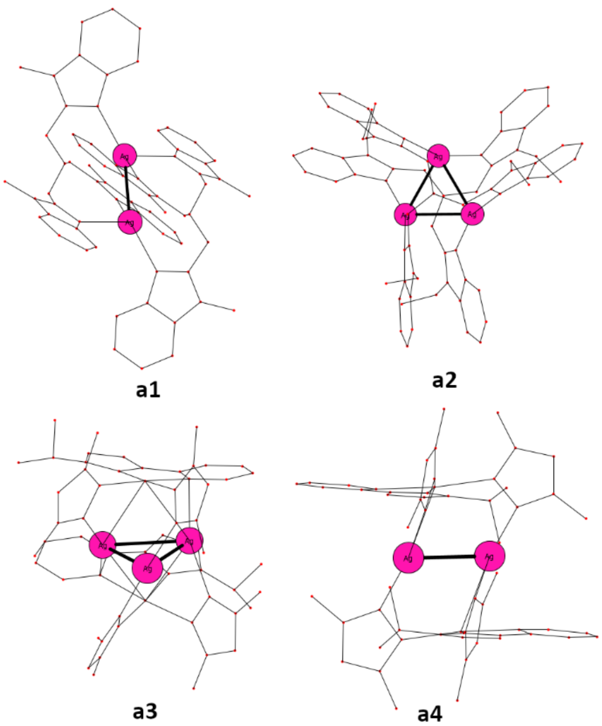

Figure S2. Schematic representation of the **a1**-**a4** systems containing Ag_2^{2+} and Ag_3^{3+} clusters synthesized in earlier studies.

Table S7. Selected bond orders for previously synthesized systems containing Ag_2^{2+} and Ag_3^{3+} clusters.

system	Wiberg index	Mayer index	Fuzzy index
**a1**			
Ag(1)-Ag(2)	0.23	0.10	0.36
**a2**			
Ag(1)-Ag(2)	0.19	<0.05	0.22
Ag(1)-Ag(3)	0.19	<0.05	0.22
Ag(2)-Ag(3)	0.19	<0.05	0.22
**a3**			
Ag(1)-Ag(2)	0.08	<0.05	0.05
Ag(1)-Ag(3)	0.18	<0.05	0.23
Ag(2)-Ag(3)	0.18	<0.05	0.23
**a4**			
Ag(1)-Ag(2)	0.15	<0.05	0.15

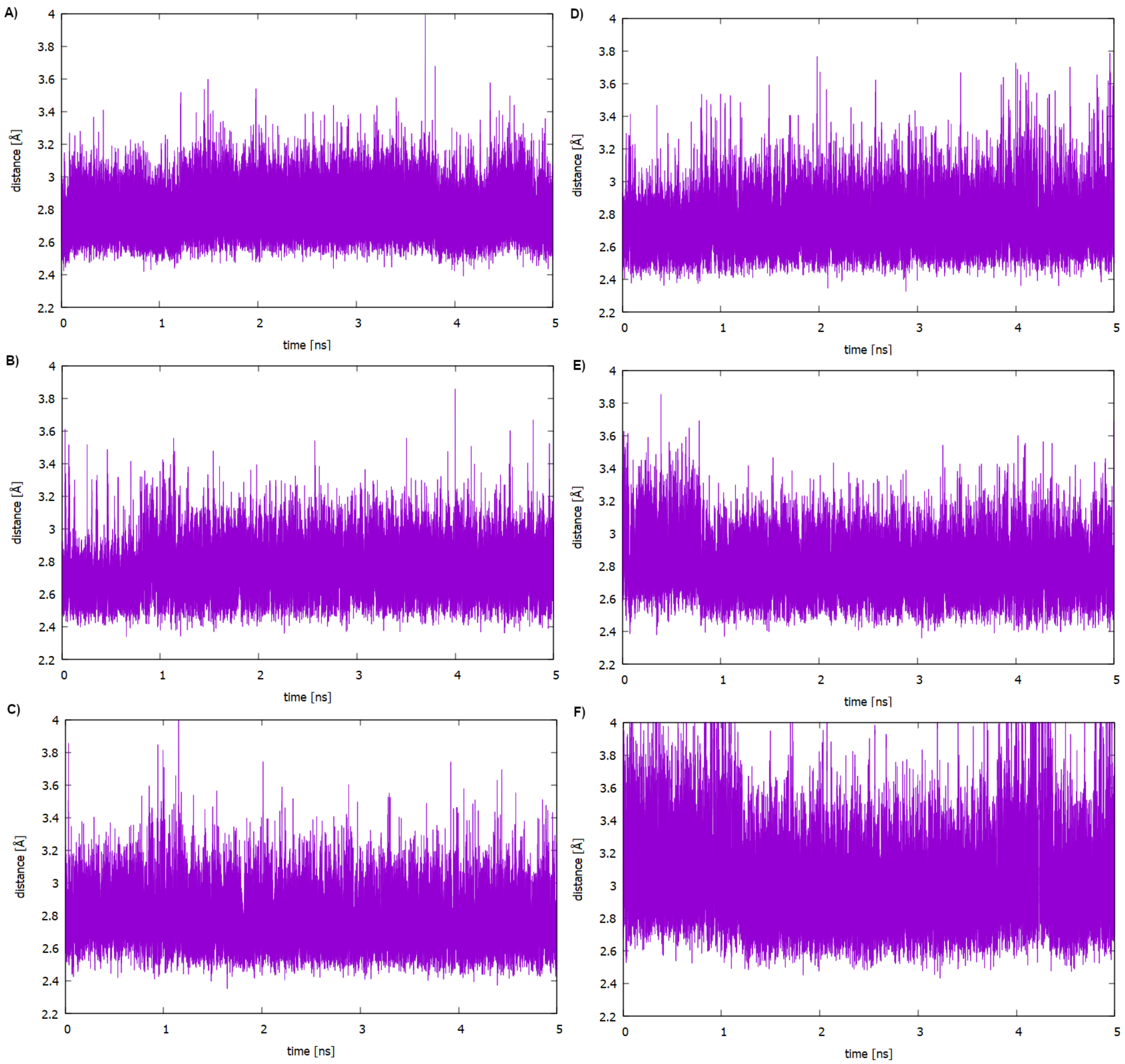

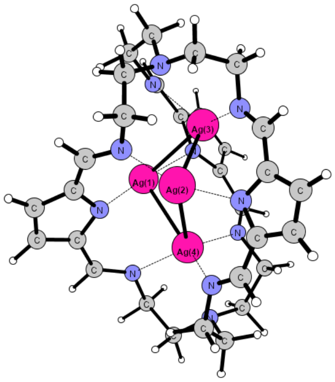

[Ag4-A⊂1]+

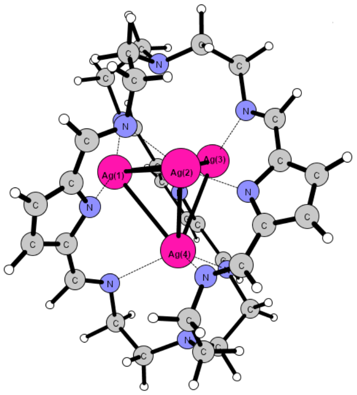

[Ag4-B⊂1]+

Figure S3. Ag-Ag bond distances during the 5 ns molecular dynamics run of the **[Ag$_4$-B⊂1]**$^+$ system; A) Ag(1)-Ag(2) distance, B) Ag(1)-Ag(3) distance, C) Ag(1)-Ag(4) distance, D) Ag(2)-Ag(3) distance, E) Ag(2)-Ag(4) distance, F) Ag(3)-Ag(4) distance. If one of the values in F) is above 3.5 Angstroms, the system is in the **[Ag$_4$-A⊂1]**$^+$ conformation; otherwise the system is in the more stable **[Ag$_4$-B⊂1]**$^+$ conformation.

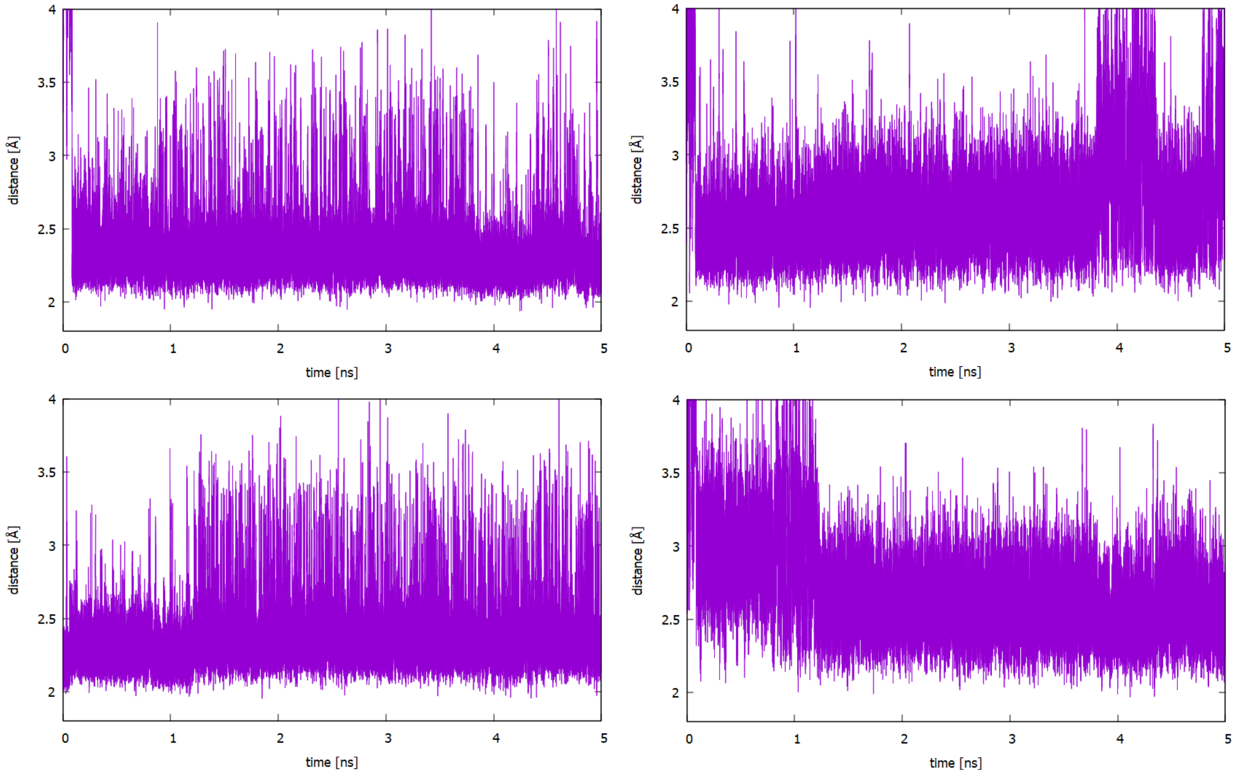

Figure S4. Ag-N bond distances during the 5 ns molecular dynamics run of the **[Ag$_4$-B⊂1]**$^+$ system between each of the four Ag atoms and the closest pyrrole N atom in the starting **[Ag$_4$-B⊂1]**$^+$ geometry.

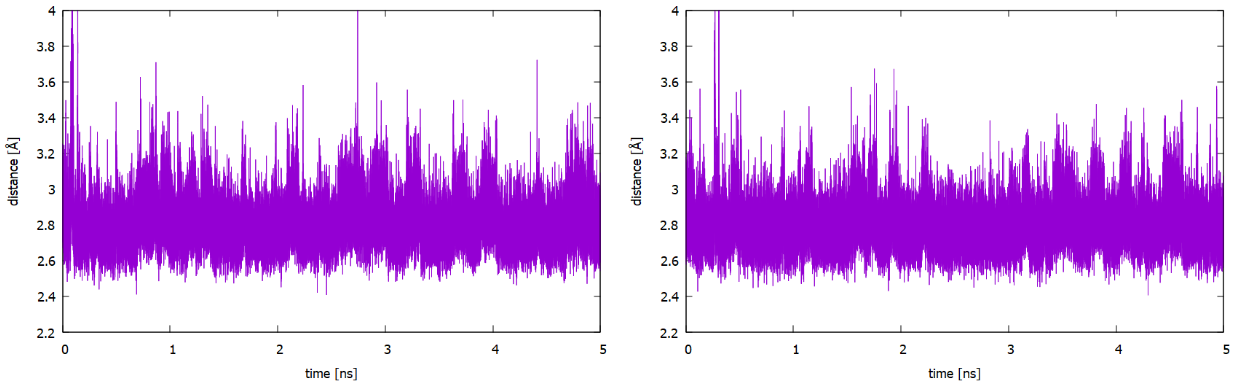

Figure S5. Ag-Ag bond distances during the 5 ns molecular dynamics run of the **[Ag_3⊂1]** system.

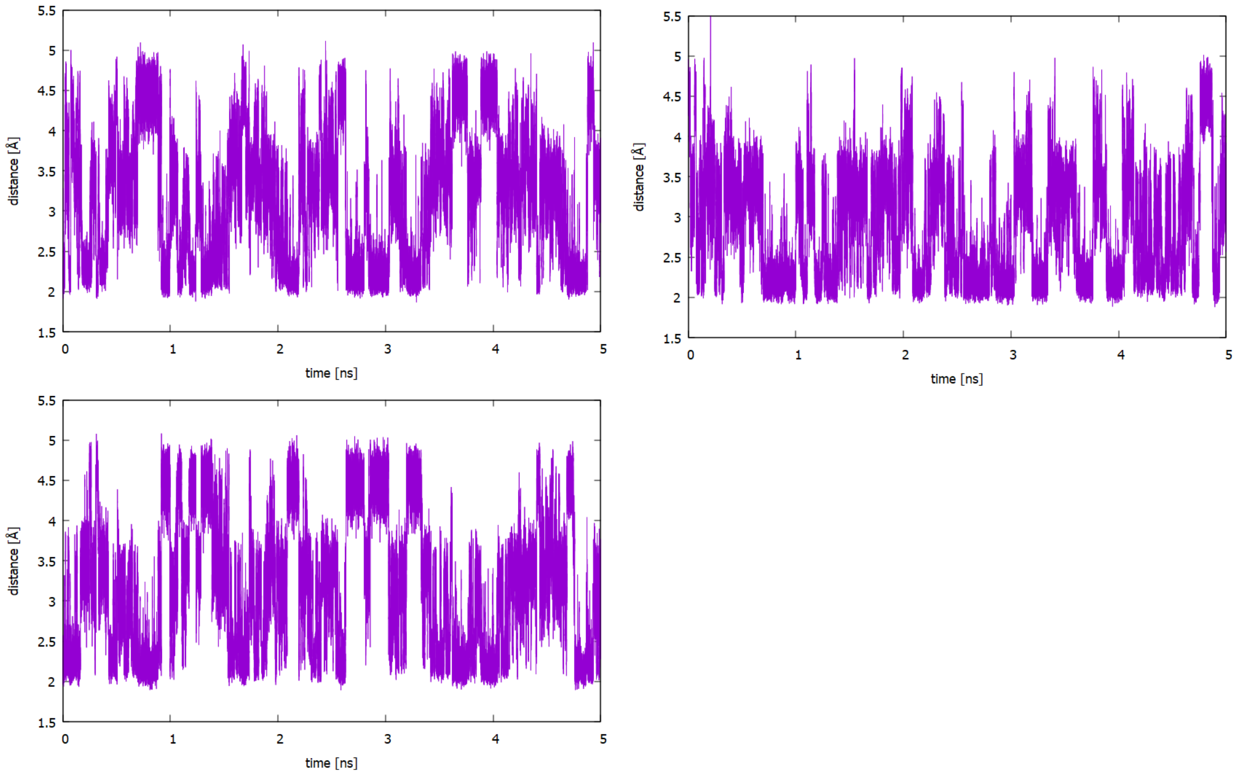

Figure S6. Ag-N bond distances during the 5 ns molecular dynamics run of the **[Ag_3⊂1]** system between one of the Ag atoms and each of the three pyrrole N atoms.

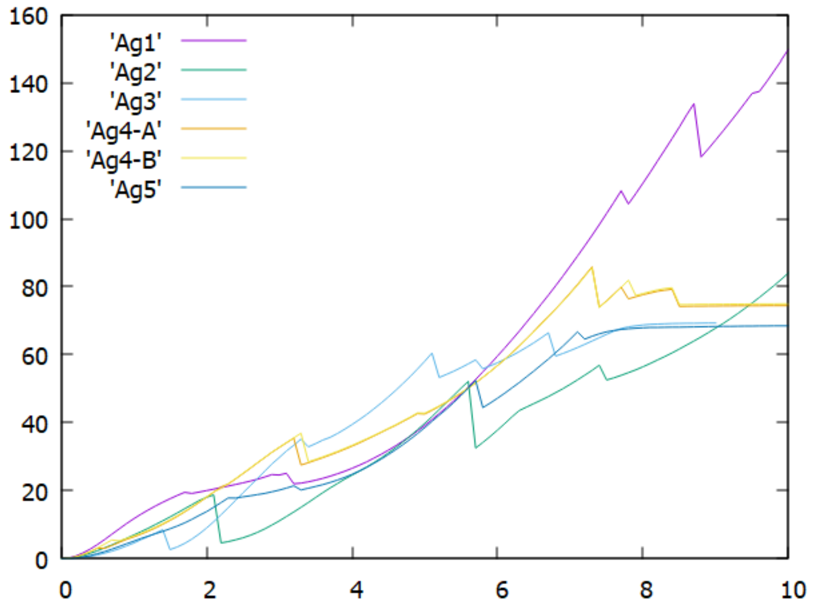

Figure S7. Potential energy surface (PES) of a single Ag^+ ion exiting the cavity of **1** for **[Ag$_n$⊂1]$^{n-3}$** systems. Distance is relative to the optimal position of each Ag^+ ion in respective complex.

Pyrrolide cages incorporating mixed-valence silver clusters

We investigated structural variations for a series of caged silver clusters, denoted as ^{m}[**Ag$_n$⊂1**]^q with n = 3 to 5. Multiplicity *m* was varied from singlet to triplet or from doublet to quartet as appropriate, and charge *q* ranges from (n–3)+ to 3–. Geometries were optimized in CH_3CN, 1-propanol (1-Pr(OH)), CH_2Cl_2 (DCM) or in the gas phase. Table S8 summarizes the results for ^{m}[Ag$_5$⊂1]^q. Each row corresponds to a geometry optimization at (PCM) U*w*B97X-d/6-31G** ~ LANL2DZ confirmed by no imaginary frequencies. The initial geometry in all cases was ^{1}[**Ag$_n$⊂1**]$^{(n–3)+}$ in the gas phase (e.g., ^{1}[**Ag$_5$⊂**1]$^{2+}$ for the n = 5 case), unless indicated otherwise. Relative energies ΔE_{rel} and ΔG_{rel} in kcal/mol were calculated considering only species with the same charge *q* (separated by double-line borders in the table). Expected square values of S are reported along with $\langle S^2\rangle_{annih}$ to inspect spin contamination. Spin densities (S_{Ag}) and Mulliken charges (q_{Ag}) in each Ag atom were added to account for total values for the metal clusters. Even

though S_{Ag} and q_{Ag} may be inaccurate due to spin contamination and methodological aspects, respectively, these are only considered as a reference point. The root-mean-square deviation (RMSD) indicates a change in the cluster configuration: no change, RMSD < 0.5 Å; distorted bipyramid, 0.0 < RMSD < 0.5 Å; and square pyramid, RMSD > 1.0 Å.

Table S8. Energy comparisons (kcal/mol), electronic properties, and structural characterization of ^{m}[**Ag$_5$⊂1**]^{q} structures. In bold the most stabilized structure for a given charge q.

Species	phase	ΔE_{rel}	ΔG_{rel}	$<S^2>$	$<S^2>_{annih}$	Σ S_{Ag} (|e|)	Σ q_{Ag} (|e|)	RMSD (Å)	Ag_5 cluster geometry
$^{1}[Ag_5⊂1]^{2+}$	CH_3CN	**0.0**	**0.0**	0.00	0.00	0.00	1.92	0.06	**trigonal bipyramidal**
	1-Pr(OH)	3.3	3.6	0.00	0.00	0.00	1.90	0.06	
	DCM	12.5	13.4	0.00	0.00	0.00	1.85	0.05	
	gas	131.2	131.3	-	-	-	1.57	0.00	
$^{3}[Ag_5⊂1]^{2+}$	CH_3CN	50.8	47.5	2.03	2.00	0.02	1.89	0.12	trigonal bipyramidal
	1-Pr(OH)	73.8	68.2	2.03	2.00	0.66	1.41	0.25	
	DCM	81.8	77.1	2.03	2.00	0.66	1.38	0.24	
	gas	213.6	210.0	2.03	2.00	0.10	1.61	0.04	
$^{2}[Ag_5⊂1]^{1+}$	CH_3CN	**0.0**	**0.0**	0.76	0.75	0.67	1.22	0.23	**trigonal bipyramidal**
	1-Pr(OH)†	1.1	1.2	0.76	0.75	0.67	1.22	0.23	
	DCM†	4.3	4.7	0.76	0.75	0.66	1.20	0.23	
	gas	42.6	42.5	0.76	0.75	0.65	1.08	0.17	
$^{4}[Ag_5⊂1]^{1+}$	CH_3CN	69.3	68.0	3.79	3.75	0.06	1.62	0.17	trigonal bipyramidal
	1-Pr(OH)	70.7	69.6	3.79	3.75	0.06	1.61	0.17	
	DCM	74.4	73.6	3.79	3.75	0.06	1.58	0.16	
	gas	93.2	90.8	3.78	3.75	0.68	1.06	0.18	

† In these cases, geometry optimizations failed, and re-optimizations proceeded from the optimized geometry of ^{2}[**Ag$_5$⊂1**]$^{1+}$ in CH_3CN. Henceforth, re-optimizations correspond to this adjustment so that ^{m}[**Ag$_5$⊂1**]^{q} in solvent was used as the initial geometry.

The results for q = 2+ and 1+ are summarized as follows:

- No structural variation, RMSD < 0.3 Å.
- High spin configurations are highly destabilized.

- The charge 2+ in $^{1}[Ag_5{\subset}1]^{2+}$ is in the Ag_5 cluster, as expected due to the five Ag^+ counteracting the charge 3– in the cage ($\mathbf{1}^{3-}$) (ΣS_{Ag} = 0.0 *e* and Σq_{Ag} = 1.6 to 1.9 *e*).
- The charge 1+ and one unpaired electron in $^{2}[Ag_5{\subset}1]^{1+}$ are in the Ag_5 cluster, as expected due to the $4Ag^+$ plus Ag^0 configuration that counteracts $\mathbf{1}^{3-}$ (Σq_{Ag} = 1.1 to 1.2 *e*), and the doublet electron Ag^0 confirmed by ΣS_{Ag} = 0.7 *e*.

Table S9. Energy, electronic and structural parameters for $^{m}[\mathbf{Ag_5{\subset}1}]^{q}$ with q = 0 to 2–.

Species	phase	Δ*E*$_{rel}$	Δ*G*$_{rel}$	$<S^2>$	$<S^2>_{annih}$	Σ S_{Ag} (|*e*|)	Σ q_{Ag} (|*e*|)	RMSD (Å)	Ag_5 cluster geometry
$^{1}[Ag_5{\subset}1]^{0}$	CH_3CN	**0.0**	**0.0**	0.00	0.00	0.00	0.60	1.11	**square pyramidal**
	1-Pr(OH)	1.0	0.9	0.00	0.00	0.00	0.61	1.11	
	DCM	3.6	3.3	0.00	0.00	0.00	0.63	1.13	
	gas	19.5	20.0	0.00	0.00	0.00	0.66	1.15	
$^{3}[Ag_5{\subset}1]^{0}$	CH_3CN	36.6	36.0	2.05	2.00	0.68	1.00	0.23	trigonal bipyramidal
	1-Pr(OH)	37.3	36.9	2.05	2.00	0.68	1.00	0.23	
	DCM	39.4	39.2	2.05	2.00	0.68	1.01	0.22	
	gas	54.6	55.3	2.05	2.00	0.68	0.98	0.20	
$^{2}[Ag_5{\subset}1]^{1-}$	CH_3CN	**0.0**	**0.0**	0.80	0.75	0.03	0.36	0.58	**distorted trigonal bipyramidal**
	1-Pr(OH)	1.9	0.0	0.80	0.75	0.03	0.38	0.58	distorted trigonal bipyramidal
	DCM†	7.1	5.1	0.80	0.75	0.03	0.41	0.59	distorted trigonal bipyramidal
	gas†	51.0	48.8	0.80	0.75	0.04	0.48	0.57	distorted trigonal bipyramidal
	gas	68.6	68.0	1.88*	1.24*	0.07	1.22	0.19	trigonal bipyramidal
$^{4}[Ag_5{\subset}1]^{1-}$	CH_3CN	24.4	22.6	3.85	3.75	0.74	0.71	0.22	trigonal bipyramidal
	1-Pr(OH)†	26.1	24.4	3.85	3.75	0.74	0.72	0.22	
	DCM	30.9	29.2	3.85	3.75	0.74	0.75	0.22	
	gas	68.7	67.7	3.89	3.76	0.11	1.22	0.19	
$^{1}[Ag_5{\subset}1]^{2-}$	CH_3CN	11.8	13.1	0.00	0.00	0.00	-0.16	0.77	distorted trigonal bipyramidal
	1-Pr(OH)†	16.9	18.3	0.00	0.00	0.00	-0.13	0.75	
	DCM	30.6	32.3	0.00	0.00	0.00	-0.05	0.61	
	gas	158.9	156.3	0.00	0.00	0.00	0.14	0.60	
$^{3}[Ag_5{\subset}1]^{2-}$	CH_3CN	**0.0**	**0.0**	2.09	2.00	0.18	-0.01	0.77	**distorted trigonal bipyramidal**
	1-Pr(OH)†	4.8	4.8	2.09	2.00	0.17	0.03	0.77	
	DCM†	17.8	18.4	2.09	2.00	0.16	0.10	0.78	
	gas†	144.8	141.9	2.10	2.00	0.13	0.33	0.73	

The results for q = 0, 1– and 2– suggest that structural variations in the Ag_5 cluster are related to changes in q rather than changes in the phase.

- ^{1}[**Ag_5⊂1**]^{0} exhibits a square pyramidal Ag_5 cluster, RMSD = 1.1 Å, and the triplet state is highly destabilized. In this square pyramidal cluster, the $3Ag^{+}$ plus $2Ag^{0}$ configuration confirms a closed-shell singlet state (ΣS_{Ag} = 0.0 e), but each Ag atom transferred to the cage a small amount of charge (< 0.3 e) as indicated by Σq_{Ag} = 0.6 e.
- The Ag_5 cluster in ^{2}[**Ag_5⊂1**]$^{1-}$ displays a structure between the initial trigonal bipyramid and square pyramid, RMSD = 0.6 Å, and the quartet state is highly destabilized. In this case, ΣS_{Ag} = 0.0 e suggests that the doublet electron is localized in the cage, where a partial charge was transferred from the Ag_5 cluster to the cage (Σq_{Ag} = 0.3 to 0.5 e). Only ^{2}[**Ag_5⊂1**]$^{1-}$ in gas phase could be optimized in trigonal bipyramidal geometry, yet it is spin-contaminated and destabilized.
- The Ag_5 cluster in ^{1}[**Ag_5⊂1**]$^{2-}$ also exhibits a distorted bipyramid, RMSD = 0.6 to 0.8 Å, but the triplet state resulted more stabilized than the closed-shell state, probably because of the stabilizing exchange of the two unpaired electrons related to the 2– charge. These two unpaired electrons are located in the cage for both the triplet and singlet states due to ΣS_{Ag} and $\Sigma q_{Ag} < |0.2|$ e, indicating an Ag^{+} plus $4Ag^{0}$ configuration counteracting **1**$^{3-}$.

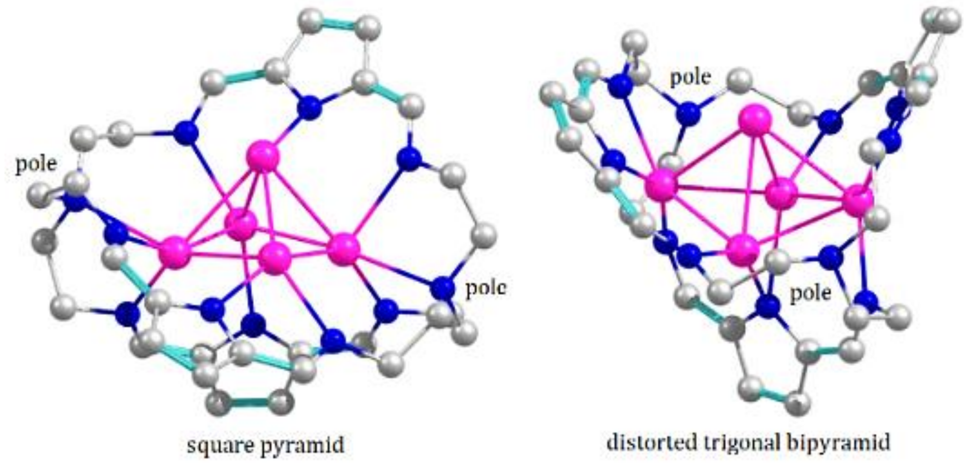

Figure S8. Structural conformations of the mixed-valence Ag_5 cluster in ^{m}[**Ag₅⊂1**]^{q}.

Table S10. Energy, electronic and structural parameters for ^{m}[**Ag₅⊂1**]^{q} with q = 3–.

species	*phase*	Δ*E_rel*	Δ*G_rel*	<S²>	<S²>*annih*	Σ S_Ag (*e*)	Σ q_Ag (*e*)	RMSD (Å)	Ag₅ cluster geometry
^{2}[Ag₅⊂1]$^{3-}$	CH₃CN†	**0.0**	**0.0**	1.88	1.26	-0.01	-0.21	1.13	**square pyramidal**
	CH₃CN	5.8	6.7	1.89	1.27	0.14	-0.20	0.90	distorted trigonal bipyramidal
	1-Pr(OH)	8.7	9.2	1.88	1.27	0.10	-0.16	1.12	square pyramidal
	DCM	40.9	44.3	1.92	1.38	0.09	-0.13	0.50	trigonal bipyramidal
	gas								*failed* (*two attempts*)
^{4}[Ag₅⊂1]$^{3-}$	CH₃CN†	10.9	11.5	3.91	3.76	0.15	-0.27	0.51	distorted trigonal bipyramidal
	1-Pr(OH)	19.0	20.9	3.91	3.76	0.14	-0.22	0.51	
	DCM	40.9	43.7	3.91	3.76	0.12	-0.13	0.50	
	gas†	293.6	289.1	3.92	3.76	0.11	0.12	0.61	

Structures in the case of ^{2}[**Ag₅⊂1**]$^{3–}$ show spin contamination, and the square pyramidal configuration seems to be preferred (although this optimization failed for the gas phase). The quartet state is not spin contaminated, but it is destabilized compared with the attempts for the doublet state (except for results in DCM that seem identical). Overall, due to the spin contamination, the description of this case in terms of structural variability is inconclusive.

Table S11. Energy, electronic and structural parameters for ^{m}[**Ag$_4$⊂1**]^{q} with q = 1+ and 0.

species	*phase*	ΔE_{rel}	ΔG_{rel}	<S^2>	<S^2>$_{annih}$	Σ S_{Ag} (e)	Σ q_{Ag} (e)	RMSD (Å)	Ag_4 cluster geometry
^{1}[Ag$_4$-A⊂1]$^{1+}$	CH_3CN	6.6	6.4	0.00	0.00	0.00	1.29	0.07	rhomboidal
	1-Pr(OH)	7.8	7.7	0.00	0.00	0.00	1.29	0.06	
	DCM	11.1	11.5	0.00	0.00	0.00	1.27	0.06	
	gas	48.5	48.4	-	-	-	1.16	0.00	
^{1}[Ag$_4$-B⊂1]$^{1+}$	CH_3CN	**0.0**	**0.0**	0.00	0.00	0.00	1.30	0.04	**trigonal pyramidal**
	1-Pr(OH)	1.2	1.3	0.00	0.00	0.00	1.29	0.03	
	DCM	4.6	4.9	0.00	0.00	0.00	1.28	0.03	
	gas	42.2	42.7	-	-	-	1.18	0.00	
^{3}[Ag$_4$-A⊂1]$^{1+}$	CH_3CN	65.6	59.0	2.05	2.00	0.75	0.83	0.67	trigonal pyramidal
	1-Pr(OH)	53.2	50.0	2.03	2.00	0.01	1.27	0.47	
	DCM	56.4	53.5	2.03	2.00	0.01	1.26	0.46	
	gas	92.5	89.6	2.03	2.00	0.01	1.17	0.49	
^{3}[Ag$_4$-B⊂1]$^{1+}$	CH_3CN	74.3	69.3	2.01	2.00	0.72	0.76	0.23	trigonal pyramidal
	1-Pr(OH)	75.7	70.7	2.01	2.00	0.72	0.76	0.23	
	DCM†	79.8	74.9	2.01	2.00	0.72	0.77	0.23	
	gas	92.4	89.8	2.03	2.00	0.01	1.17	0.06	

species	*phase*	ΔE_{rel}	ΔG_{rel}	<S^2>	<S^2>$_{annih}$	Σ S_{Ag} (e)	Σ q_{Ag} (e)	RMSD (Å)	Ag_4 cluster geometry
^{2}[Ag$_4$-A⊂1]^{0}	CH_3CN	7.5	8.2	0.79	0.75	0.05	1.00	0.21	rhomboidal
	1-Pr(OH)	8.4	8.9	0.79	0.75	0.05	1.00	0.21	rhomboidal
	DCM	4.5	5.0	0.75	0.75	0.74	0.73	0.61	trigonal pyramidal
	gas	21.8	24.6	0.80	0.75	0.03	1.04	0.52	trigonal pyramidal
^{2}[Ag$_4$-B⊂1]^{0}	CH_3CN	**0.0**	**0.0**	0.76	0.75	0.73	0.65	0.34	**trigonal pyramidal**
	1-Pr(OH)	0.8	0.9	0.76	0.75	0.73	0.66	0.34	
	DCM	3.0	3.1	0.76	0.75	0.73	0.67	0.34	
	gas	21.8	24.3	0.80	0.75	0.03	1.04	0.10	
^{4}[Ag$_4$-A⊂1]^{0}	CH_3CN	61.0	58.9	3.82	3.75	0.06	1.00	0.15	rhomboidal
	1-Pr(OH)	55.6	53.4	3.78	3.75	0.78	0.66	0.69	trigonal pyramidal
	DCM	64.3	62.5	3.82	3.75	0.06	1.01	0.16	rhomboidal
	gas	78.8	76.6	3.82	3.75	0.06	0.98	0.17	rhomboidal
^{4}[Ag$_4$-B⊂1]^{0}	CH_3CN	53.4	51.0	3.78	3.75	0.74	0.61	0.37	trigonal pyramidal
	1-Pr(OH)	54.2	51.8	3.78	3.75	0.74	0.62	0.37	
	DCM	58.1	57.4	3.82	3.75	0.03	1.04	0.10	
	gas	73.8	73.3	3.82	3.75	0.04	1.03	0.10	

The results for q = 1+ and 0 in the case of four Ag atoms are summarized as follows:

- RMSD for [**Ag_4A⊂1**] and [**Ag_4B⊂1**] was calculated comparing the initial rhomboidal and trigonal pyramidal geometries in the gas phase, respectively. In general, there are no structural variations, RMSD < 0.4 Å, but in some cases the rhomboidal [**Ag_4A⊂1**] turned into trigonal pyramid as indicated by RMSD = 0.5 to 0.7 Å. Indeed, the trigonal pyramidal configuration is energetically more stable than the rhomboidal counterpart.
- High spin configurations are highly destabilized.
- The charge 1+ in both ^{1}[**Ag_4A⊂1**]$^{1+}$ and ^{1}[**Ag_4B⊂1**]$^{1+}$ is in the Ag_4 cluster (Σq_{Ag} = 1.3 e), as expected due to the four Ag^+ (ΣS_{Ag} = 0.0 e) counteracting **1**$^{3-}$.
- In the case of <u>rhomboidal</u> ^{2}[**Ag_4A⊂1**]^0, the assumed 3Ag^+ plus Ag^0 configuration is actually 4Ag^+ counteracting **1**$^{4-}$; that is, the doublet electron due to Ag^0 was transferred to the cage, as indicated by ΣS_{Ag} =0.0 e and Σq_{Ag} = 1.0 e. A similar result was found for trigonal pyramidal ^{2}[**Ag_4A⊂1**]^0 and ^{2}[**Ag_4B⊂1**]^0 in the gas phase.
- In the case of <u>trigonal pyramidal</u> ^{2}[**Ag_4A⊂1**]^0 and ^{2}[**Ag_4B⊂1**]^0 in solution, the assumed 3Ag^+ plus Ag^0 configuration does retain the doublet Ag^0 electron (ΣS_{Ag} = 0.7 e), but each Ag^+ transferred ca. 0.2 e to the cage, as indicated by and Σq_{Ag} = 0.7 e.

Table S12. Energy, electronic and structural parameters for $^{m}[\mathbf{Ag_4{\subset}1}]^{q}$ with q = 1–.

species	*phase*	ΔE_{rel}	ΔG_{rel}	$\langle S^2 \rangle$	$\langle S^2 \rangle_{annih}$	Σ S_{Ag} (e)	Σ q_{Ag} (e)	RMSD (Å)	Ag_4 cluster geometry
$^{1}[Ag_4\text{-}A{\subset}1]^{1-}$	CH_3CN	49.8	51.5	1.10	0.76	-0.02	0.80	0.13	rhomboidal
	$CH_3CN^{\dagger}$	**0.0**	**0.0**	0.00	0.00	0.00	0.11	1.10	**trigonal pyramidal (1)**
	1-Pr(OH)†	2.6	2.7	0.00	0.00	0.00	0.13	1.11	trigonal pyramidal (1)
	DCM	9.1	8.7	0.00	0.00	0.00	0.18	1.11	trigonal pyramidal (1)
	gas†	57.5	56.2	0.00	0.00	0.00	0.34	1.10	trigonal pyramidal (1)
	gas	74.4	74.2	0.00	0.00	0.00	0.22	0.90	trigonal pyramidal (2)
$^{1}[Ag_4\text{-}B{\subset}1]^{1-}$	CH_3CN								*failed*
	1-Pr(OH)								*failed*
	DCM								*failed*
	gas	74.4	74.3	0.00	0.00	0.00	0.22	0.78	trigonal pyramidal (2)
$^{3}[Ag_4\text{-}A{\subset}1]^{1-}$	CH_3CN	49.8	50.9	2.10	2.00	0.04	0.80	0.13	rhomboidal
	$CH_3CN^{\dagger}$	42.3	44.4	2.10	2.00	0.04	0.82	0.50	trigonal pyramidal'
	1-Pr(OH)†	44.3	46.7	2.10	2.00	0.04	0.83	0.50	trigonal pyramidal'
	DCM	62.1	68.4	2.06	2.00	0.09	0.83	0.52	trigonal pyramidal'
	gas	94.2	95.7	2.10	2.00	0.04	0.92	0.53	trigonal pyramidal'
$^{3}[Ag_4\text{-}B{\subset}1]^{1-}$	CH_3CN	42.3	43.7	2.10	2.00	0.04	0.82	0.12	trigonal pyramidal
	1-Pr(OH)	44.4	46.0	2.10	2.00	0.04	0.83	0.12	
	DCM	49.6	51.9	2.10	2.00	0.03	0.85	0.13	
	gas	94.2	95.3	2.10	2.00	0.04	0.92	0.12	

The results for q = 1– are summarized as follows:

- High spin configurations are highly destabilized.
- The rhomboidal $^{1}[\mathbf{Ag_4A{\subset}1}]^{1-}$ is highly destabilized so that the optimizations led to the trigonal pyramid. Unexpectedly, geometry optimizations of the trigonal pyramidal $^{1}[\mathbf{Ag_4B{\subset}1}]^{1-}$ didn't converge. Since $^{1}[\mathbf{Ag_4A{\subset}1}]^{1-}$ converged into the trigonal pyramid, $^{1}[\mathbf{Ag_4B{\subset}1}]^{1-}$ was not re-optimized.
- Considering all cases, four different trigonal pyramidal configurations were identified, denoted with no mark (RMSD = 0.10 Å), 'apostrophe (RMSD = 0.50 Å), (1) (RMSD = 1.1 Å), and (2) (RMSD = 0.8 – 0.9 Å).

- In the case of the trigonal pyramidal (1) optimized from ^{1}[**Ag$_4$A⊂1**]$^{1-}$, the charge 1– is in the cage, as expected due to the two 2Ag^{+} counteracting **1**$^{3-}$. In fact, together with the other 2Ag^{0} it corresponds to a closed-shell system with a small amount of charge transferred to the cage, as indicated by $\Sigma S_{Ag} = 0.0\ e$ and $\Sigma q_{Ag} < 0.4\ e$. In contrast, we observe that the rhomboidal structure (first entry) is a doublet that may contain spin contamination.

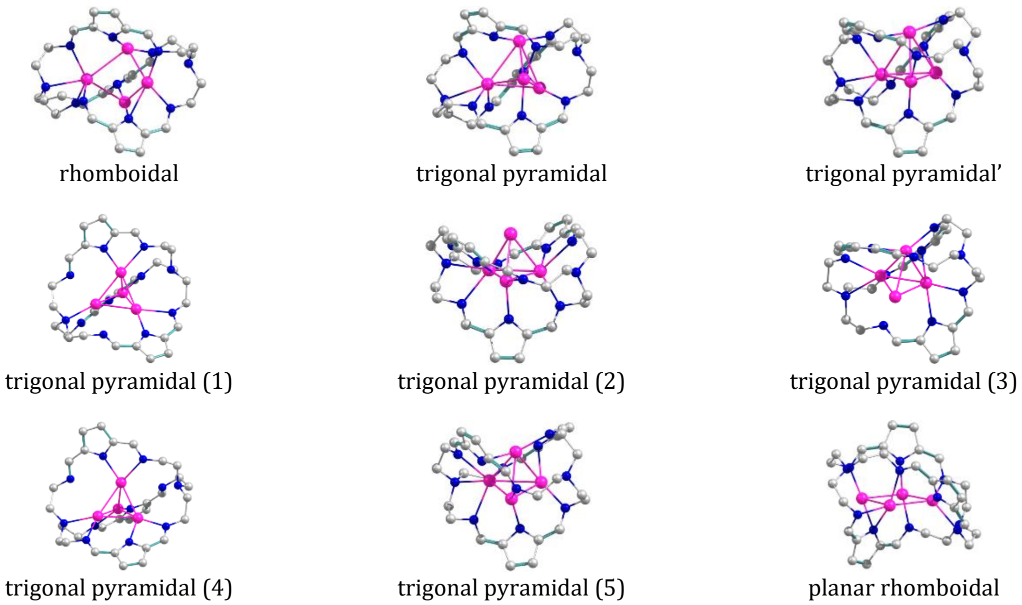

Figure S9. Structural conformations of the mixed-valence Ag$_4$ cluster in ^{m}[**Ag$_4$⊂1**]^{q}.

Table S13. Energy, electronic and structural parameters for ^{m}[**Ag$_4$⊂1**]^{q} with q = 2–.

species	*phase*	ΔE_{rel}	ΔG_{rel}	<S^2>	<S^2>$_{annih}$	Σ S_{Ag} (e)	Σ q_{Ag} (e)	RMSD (Å)	Ag$_4$ cluster geometry
^{2}[Ag$_4$-A⊂1]$^{2-}$	CH$_3$CN								*failed*
	1-Pr(OH)								*failed*
	DCM								*failed*
	gas	135.1	131.4	0.81	0.75	0.02	0.04	0.67	trigonal pyramidal (3)
^{2}[Ag$_4$-B⊂1]$^{2-}$	CH$_3$CN	0.0	0.5	1.90	1.30	0.01	0.61	0.08	trigonal pyramidal
	1-Pr(OH)	4.2	4.4	1.90	1.30	0.01	0.62	0.09	trigonal pyramidal
	DCM	15.8	17.0	1.90	1.31	0.01	0.66	0.11	trigonal pyramidal
	gas	135.1	131.6	0.81	0.75	0.02	0.04	0.86	trigonal pyramidal (3)
^{4}[Ag$_4$-A⊂1]$^{2-}$	CH$_3$CN	6.9	5.7	3.86	3.75	0.80	0.21	0.64	trigonal pyramidal (3)
	1-Pr(OH)								*failed*
	DCM								*failed*
	gas	139.4	137.2	3.90	3.76	0.04	0.82	0.47	trigonal pyramidal (3)
^{4}[Ag$_4$-B⊂1]$^{2-}$	CH$_3$CN	**0.0**	**0.0**	3.90	3.76	0.04	0.61	0.08	**trigonal pyramidal**
	1-Pr(OH)	4.2	4.1	3.90	3.76	0.04	0.62	0.09	
	DCM	15.8	16.6	3.90	3.76	0.04	0.66	0.11	
	gas	139.4	137.4	3.90	3.76	0.04	0.82	0.08	

The results for q = 2– are summarized as follows:

- The rhomboidal ^{2}[**Ag$_4$A⊂1**]$^{2–}$ could not be optimized, so that the trigonal pyramid ^{2}[**Ag$_4$B⊂1**]$^{2–}$ remains the equilibrium geometry.
- Structures ^{2}[**Ag$_4$B⊂1**]$^{2–}$ in solution exhibit spin contamination and, unexpectedly, the other parameters are nearly identical to those calculated for the quartet state. This suggests that the doublet state will not be formed, except for the gas-phase ^{2}[**Ag$_4$B⊂1**]$^{2–}$.
- The quartet-spin structures are not spin contaminated. The initially assumed Ag^+ plus 3Ag^0 configuration in ^{4}[**Ag$_4$B⊂1**]$^{2–}$ counteracting **1**$^{3–}$ apparently was turned into 2Ag^+ plus 2Ag^0 (ΣS_{Ag} = 0.0 e) that counteracts **1**$^{4–}$ instead **1**$^{3–}$. That is, each Ag$_4$ transferred 0.15 – 0.20 e to the cage: Σq_{Ag} = 0.6 to 0.8 e. The quartet electrons are therefore in the cage.

Table S14. Energy, electronic and structural parameters for ^{m}[**Ag₄⊂1**]^q with q = 3–.

species	*phase*	ΔE_{rel}	ΔG_{rel}	<S²>	<S²>$_{annih}$	Σ S_{Ag} (e)	Σ q_{Ag} (e)	RMSD (Å)	Ag_4 cluster geometry
^{1}[Ag_4-A⊂1]$^{3-}$	CH_3CN								*failed*
	1-Pr(OH)								*failed*
	DCM								*failed*
	gas	350.1	348.6	0.00	0.00	0.00	0.50	0.62	planar rhomboidal
^{1}[Ag_4-B⊂1]$^{3-}$	CH_3CN								*failed*
	1-Pr(OH)	49.0	53.3	1.10	0.79	0.00	0.34	0.14	trigonal pyramidal
	DCM								*failed*
	gas								*failed*
^{3}[Ag_4-A⊂1]$^{3-}$	CH_3CN	**0.0**	**0.0**	2.09	2.00	0.13	-0.37	1.16	**trigonal pyramidal (4)**
	1-Pr(OH)†	9.7	9.8	2.09	2.00	0.12	-0.31	1.17	trigonal pyramidal (4)
	DCM								*failed*
	gas	332.3	330.0	3.12	2.35	-0.87	0.04	0.51	trigonal pyramidal (high apex)
^{3}[Ag_4-B⊂1]$^{3-}$	CH_3CN	37.3	36.6	2.65	2.13	-0.44	-0.10	0.71	trigonal pyramidal (5)
	1-Pr(OH)	45.7	46.4	2.66	2.13	-0.45	-0.05	0.71	trigonal pyramidal (5)
	DCM								*failed*
	gas	332.1	329.0	2.10	2.00	0.02	0.62	0.16	trigonal pyramidal

Finally, rhomboidal ^{1}[**Ag₄A⊂1**]$^{3-}$ could not be optimized, except for a destabilized planar rhomboid in the gas phase. The trigonal pyramid ^{1}[**Ag₄B⊂1**]$^{3-}$ also exhibited optimization issues, and the structure in 1-propanol was the only case with an optimized geometry, but with spin contamination. The geometry in the triplet state did optimize in the trigonal pyramidal (4) structure, and ^{3}[**Ag₄A⊂1**]$^{3-}$ was highly rearranged (RMSD = 1.2 Å). The trigonal pyramidal (5) in ^{3}[**Ag₄B⊂1**]$^{3-}$also rearranged (RMSD = 0.7 Å), but it became more destabilized and with spin contamination. In general, due to the spin contamination and geometry-optimization issues, the description of this case in terms of structural variability is inconclusive.

Table S15. Energy, electronic and structural parameters for ^{m}[**Ag$_3$⊂1**]^{q} structures.

species	*phase*	Δ*E*$_{rel}$	Δ*G*$_{rel}$	<S^2>	<S^2>$_{annih}$	Σ S$_{Ag}$ (*e*)	Σ q$_{Ag}$ (*e*)	RMSD (Å)	Ag$_3$ cluster geometry
^{1}[Ag$_3$⊂1]^{0}	CH$_3$CN	**0.0**	**0.0**	0.00	0.00	0.00	0.77	0.18	**trigonal**
	1-Pr(OH)	0.9	1.0	0.00	0.00	0.00	0.77	0.17	
	DCM	3.3	3.5	0.00	0.00	0.00	0.77	0.14	
	gas	18.5	17.9	-	-	-	0.75	0.00	
^{3}[Ag$_3$⊂1]^{0}	CH$_3$CN	53.1	50.0	2.03	2.00	0.00	0.75	0.16	trigonal
	1-Pr(OH)	54.1	51.1	2.03	2.00	0.00	0.75	0.15	
	DCM	56.5	53.6	2.03	2.00	0.00	0.76	0.13	
	gas	72.7	68.3	2.03	2.00	0.01	0.74	0.13	
^{2}[Ag$_3$⊂1]$^{1-}$	CH$_3$CN	9.5	9.5	0.79	0.75	0.03	0.57	0.25	**trigonal**
	1-Pr(OH)	**0.0**	**0.0**	0.80	0.75	0.02	0.58	0.13	
	DCM	5.2	5.7	0.80	0.75	0.02	0.60	0.12	
	gas	60.6	59.3	0.80	0.75	0.03	0.61	0.14	
^{4}[Ag$_3$⊂1]$^{1-}$	CH$_3$CN	69.4	67.3	3.80	3.75	0.03	0.56	0.21	trigonal
	1-Pr(OH)	71.4	69.5	3.80	3.75	0.03	0.57	0.21	
	DCM	71.2	69.2	3.82	3.75	0.04	0.57	0.21	
	gas	102.8	100.2	3.83	3.75	0.03	0.69	0.55	
^{1}[Ag$_3$⊂1]$^{2-}$	CH$_3$CN	**0.0**	**0.0**	0.00	0.00	0.00	-0.49	1.24	**trigonal (1)**
	1-Pr(OH)	6.3	7.1	0.00	0.00	0.00	-0.47	1.16	trigonal (1)
	DCM								*failed*
	gas†	185.1	184.5	0.00	0.00	0.00	0.47	0.56	trigonal
^{3}[Ag$_3$⊂1]$^{2-}$	CH$_3$CN	37.4	37.8	2.09	2.00	0.04	0.37	0.22	trigonal
	1-Pr(OH)	45.6	48.7	2.07	2.00	0.13	0.33	0.24	
	DCM	57.3	60.5	2.07	2.00	0.12	0.37	0.25	
	gas	174.2	172.2	2.10	2.00	0.04	0.58	0.26	
^{2}[Ag$_3$⊂1]$^{3-}$	CH$_3$CN†	**0.0**	**0.0**	0.80	0.75	0.04	-0.71	1.60	**trigonal (1)**
	1-Pr(OH)								*failed*
	DCM								*failed*
	gas†	307.7	299.8	0.81	0.75	0.02	-0.29	1.46	trigonal (1)
	gas	330.2	323.4	1.90	1.33	0.01	0.48	0.42	trigonal
^{4}[Ag$_3$⊂1]$^{3-}$	CH$_3$CN	65.8	59.8	3.81	3.75	0.03	-0.68	1.18	trigonal (1)
	1-Pr(OH)†	48.4	45.4	3.85	3.75	0.80	-0.22	0.86	trigonal (1)
	DCM	68.2	70.5	3.90	3.76	0.04	0.26	0.25	trigonal
	gas†	331.9	322.4	3.88	3.76	0.82	0.12	0.92	trigonal (1)
	gas	330.2	323.1	3.91	3.76	0.04	0.48	0.43	trigonal

Results for q = 0 and 1–,

- No structural variation, RMSD < 0.3 Å.
- High spin configurations are highly destabilized.
- In the case of $^{1}[\mathbf{Ag_3{\subset}1}]^{0}$, each Ag^+ transferred additional ca. 0.25 e to the cage, since ΣS_{Ag} =0.0 e and Σq_{Ag} = 0.77 e.
- In the case of $^{2}[\mathbf{Ag_3{\subset}1}]^{1-}$, the assumed $2Ag^+$ plus Ag^0 configuration may be represented as $3Ag^+$; that is, the doublet electron due to Ag^0 was partially transferred to the cage, as indicated by ΣS_{Ag} =0.0 e and Σq_{Ag} = 0.6 e. Therefore, the charge 1– in $^{2}[\mathbf{Ag_3{\subset}1}]^{1-}$ is originated by $3Ag^+$ counteracting the cage $\mathbf{1}^{4-}$ with a doublet electron.

Results for q = 2– and 3–,

- Structural rearrangements led to the trigonal (1) geometry, RMSD > 0.8 Å.
- High spin configurations in solution are highly destabilized.
- In the case of trigonal (1) $^{1}[\mathbf{Ag_3{\subset}1}]^{2-}$ in solution, the assumed Ag^+ plus $2Ag^0$ configuration does correspond to a closed-shell system (ΣS_{Ag} =0.0 e), but the cage transferred ca. 0.25 e to each Ag^0, as indicated by Σq_{Ag} = –0.5 e. We observed a similar trend for trigonal (1) $^{2}[\mathbf{Ag_3{\subset}1}]^{3-}$ in CH_3CN.

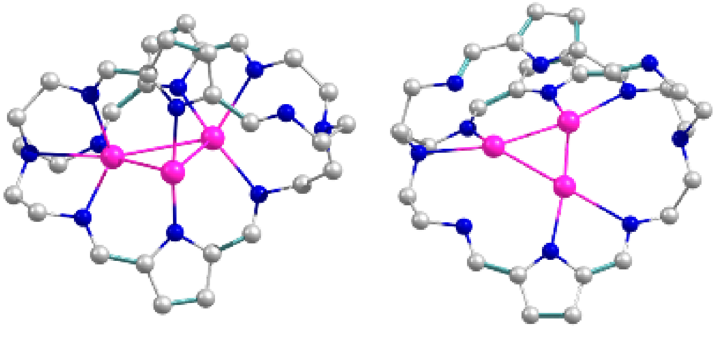

trigonal　　　　trigonal (1)

Figure S10. Structural conformations of the mixed-valence Ag_3 cluster in $^{m}[\mathbf{Ag_3{\subset}1}]^{q}$.

Time-dependent DFT UV-Vis spectra of complexes ^{m}[**Ag$_n$⊂1**]^{q}

We report TDDFT UV-Vis spectra for a series of caged silver clusters, denoted as ^{1}[**Ag$_n$⊂1**]^{q} with n = 3 to 5, and charge *q* ranging from (n–3)+ to 2–. Restricted singlet-to-singlet and unrestricted triplet-to-triplet transitions were considered. The spectra were evaluated regarding solvation or the gas phase, each case at the respective equilibrium geometry, that is:

(*phase*) TD-*w*B97X-d/6-31G** ~ LANL2DZ // (*phase*) *w*B97X-d/6-31G** ~ LANL2DZ

where *phase* stands for either PCM=solvent or the gas phase. The total number of electronic transitions was set to 100.

A. Solvent effects in ^{1}[**Ag$_3$⊂1**]^{0}

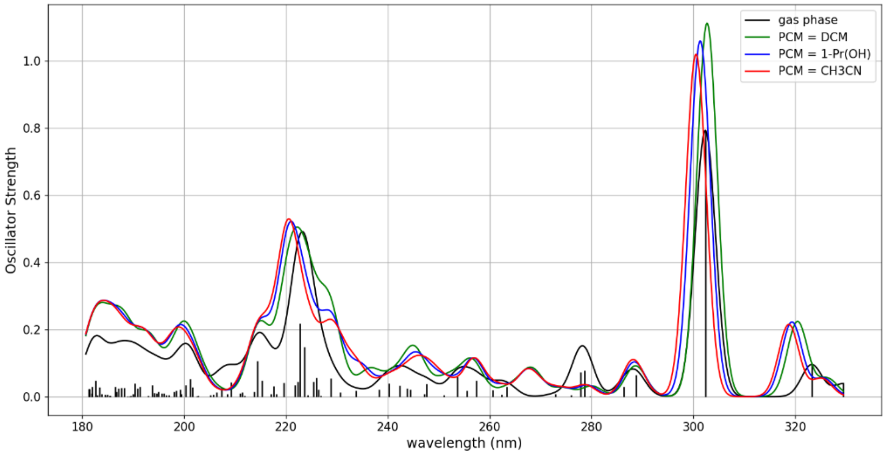

Figure S11. Effect of the environment polarization on electronic excitations in ^{1}[**Ag$_3$⊂1**]^{0}.

→ There are no transitions in the visible region. The main excitation is observed at ca. 300 nm, with HOMO to LUMO+2 as the main transition.

→ Excitation energies are blue shifted (less than 10 nm) and less intense (smaller oscillator strength) as the dielectric constant of the solvent increases. The largest variation (less than 20 nm) is observed in the 260-280 nm region.

B. Cluster-size variations: ^{1}[**Ag$_{3}$⊂1**]^{0}, ^{1}[**Ag$_{4}$⊂1**]$^{1+}$, and ^{1}[**Ag$_{5}$⊂1**]$^{2+}$

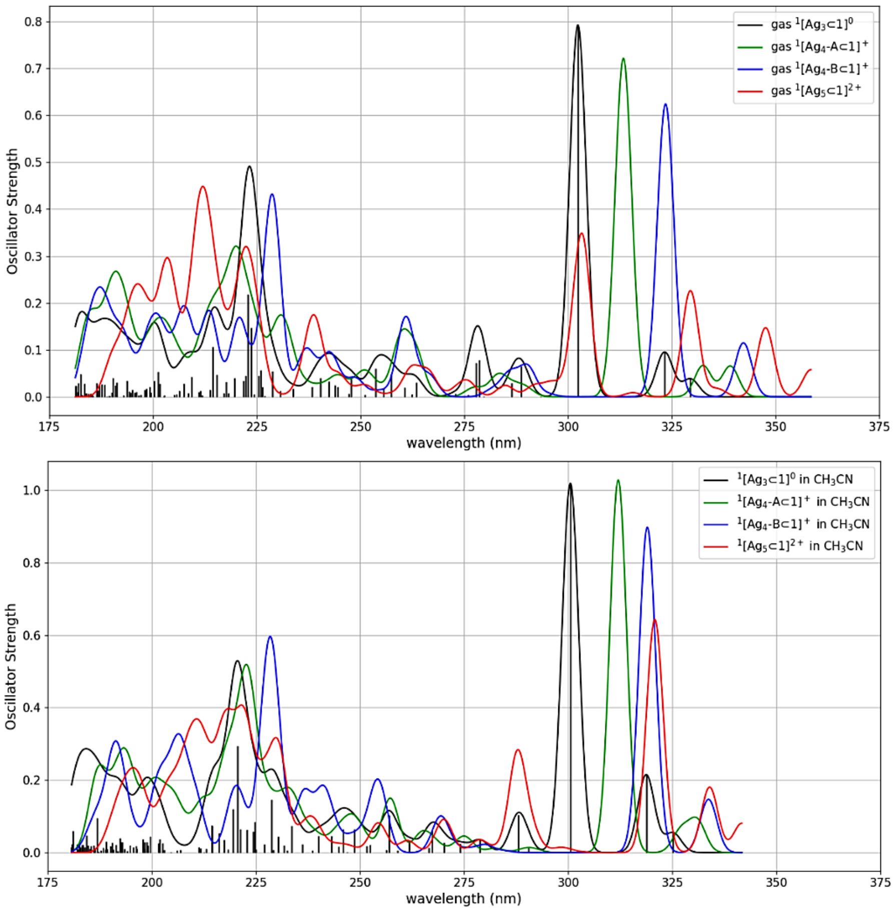

Figure S12. Effect of the Ag$_{n}$ cluster size inside the cage **1** in the gas phase and in solution.

→ The spectra exhibit variations in the shortwave UV region. On the other hand, it may be more illustrative to examine the main excitation, which occurs in the longwave UV region (300–325 nm). Considering that the solvent slightly shifts and alters intensity of a band, differences in the spectra are attributed to structural variations.

→ Accordingly, the planar configuration in trigonal ^{1}[**Ag$_{3}$⊂1**]^{0} and rhomboidal ^{1}[**Ag$_{4}$-A⊂1**]$^{1+}$ exhibit the main excitation at shorter wavelengths and more intensely compared with pyramidal ^{1}[**Ag$_{4}$-B⊂1**]$^{1+}$ and ^{1}[**Ag$_{5}$⊂1**]$^{2+}$.

C. Mixed-valence Ag_5 clusters in ^{1}[**Ag$_5$⊂1**]^q

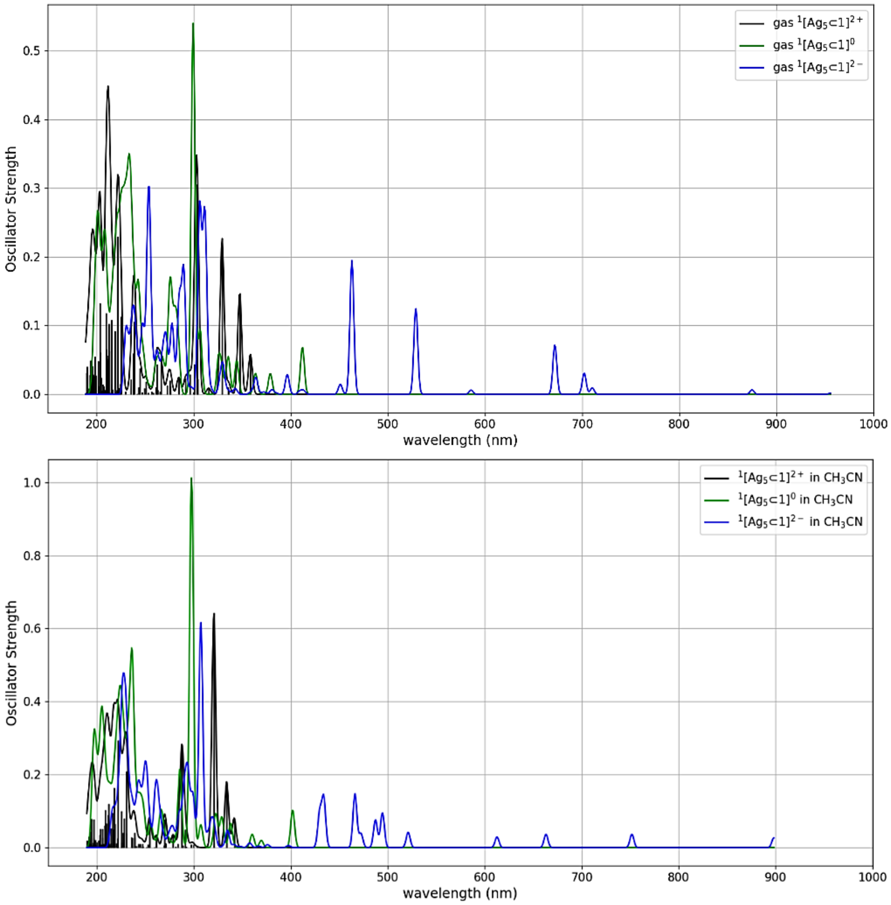

Figure S13. Comparisons of electronic excitations due to silver(I) and mixed-valence silver clusters in ^{1}[**Ag$_5$⊂1**]^q in the gas phase and in solution.

→ The introduction of metallic Ag to form mixed-valence clusters slightly alters the main excitation, but a more interesting effect is observed: excitations in the visible region! Such an effect is even more pronounced when the number of Ag^0 > number of Ag^+, for example in ^{1}[**Ag$_5$⊂1**]$^{2-}$ (four Ag^0 and one Ag^+). These colored compounds can be attributed to the presence of mixed-valence Ag clusters.

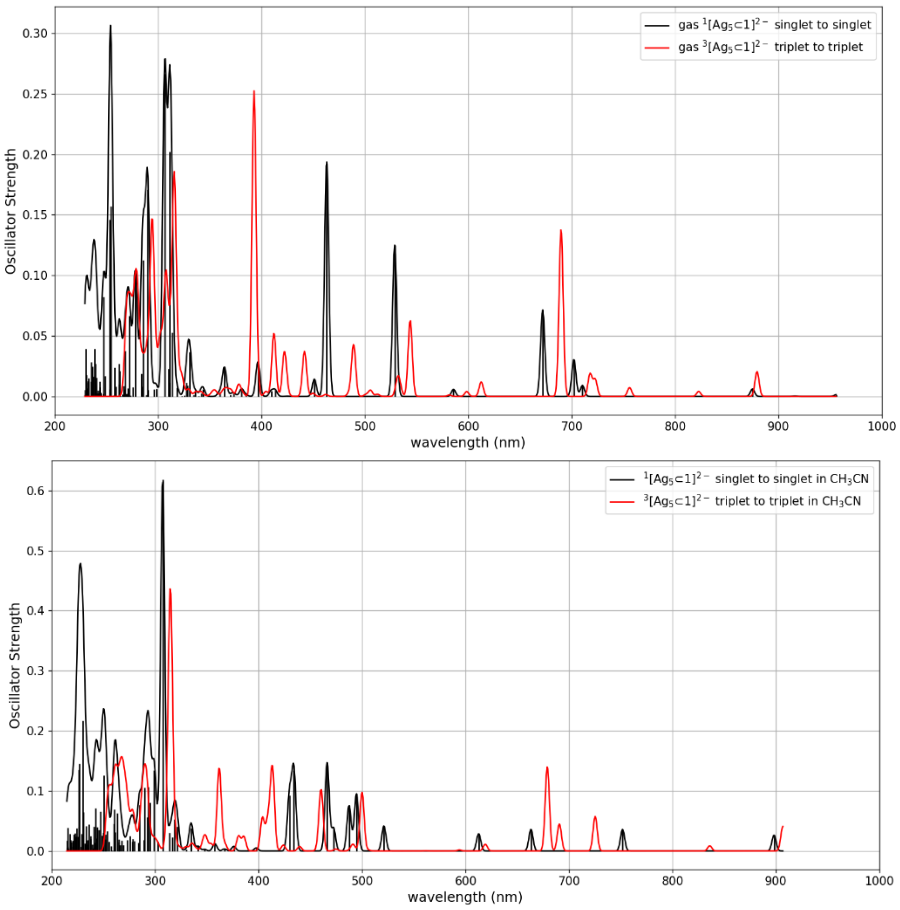

Figure S14. Comparisons of electronic excitations due to singlet-to-single transitions in ^{1}[**Ag$_5$⊂1**]$^{2–}$ and triplet-to-triplet transitions in ^{3}[**Ag$_5$⊂1**]$^{2–}$ in the gas phase and in solution.

→ The triplet spin state ^{3}[**Ag$_5$⊂1**]$^{2–}$ is more stable than the singlet state ^{1}[**Ag$_5$⊂1**]$^{2–}$. Therefore, we also calculated triplet-triplet transitions, but only for this case. However, the same observation persisted: These colored compounds may be attributed to the presence of mixed-valence Ag clusters.

D. Mixed-valence Ag_4 clusters in ^{1}[**Ag_4⊂1**]^q

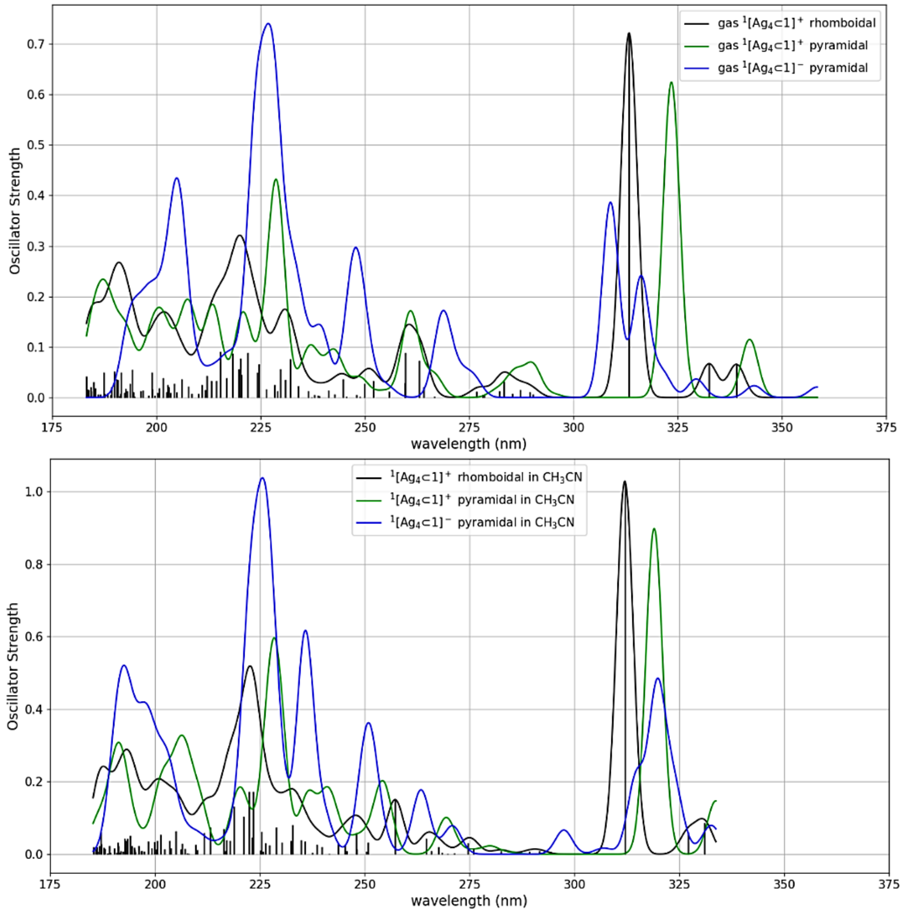

Figure S15. Comparisons of electronic excitations due to silver(I) and mixed-valence silver clusters in ^{1}[**Ag_4⊂1**]^q in the gas phase and in solution.

→ Results reported for the mixed-valence Ag cluster in ^{1}[**Ag_4⊂1**]$^{1-}$ suggest that absorptions in the visible region is driven by the condition of Ag^0 > Ag^+, which is not met in ^{1}[**Ag_4⊂1**]$^{1-}$ (2Ag^0 and 2Ag^+), unlike the previous case ^{1}[**Ag_5⊂1**]$^{2-}$ (4Ag^0 and Ag^+).

E. Mixed-valence Ag_3 clusters in ^{1}[**Ag_3⊂1**]^{q}

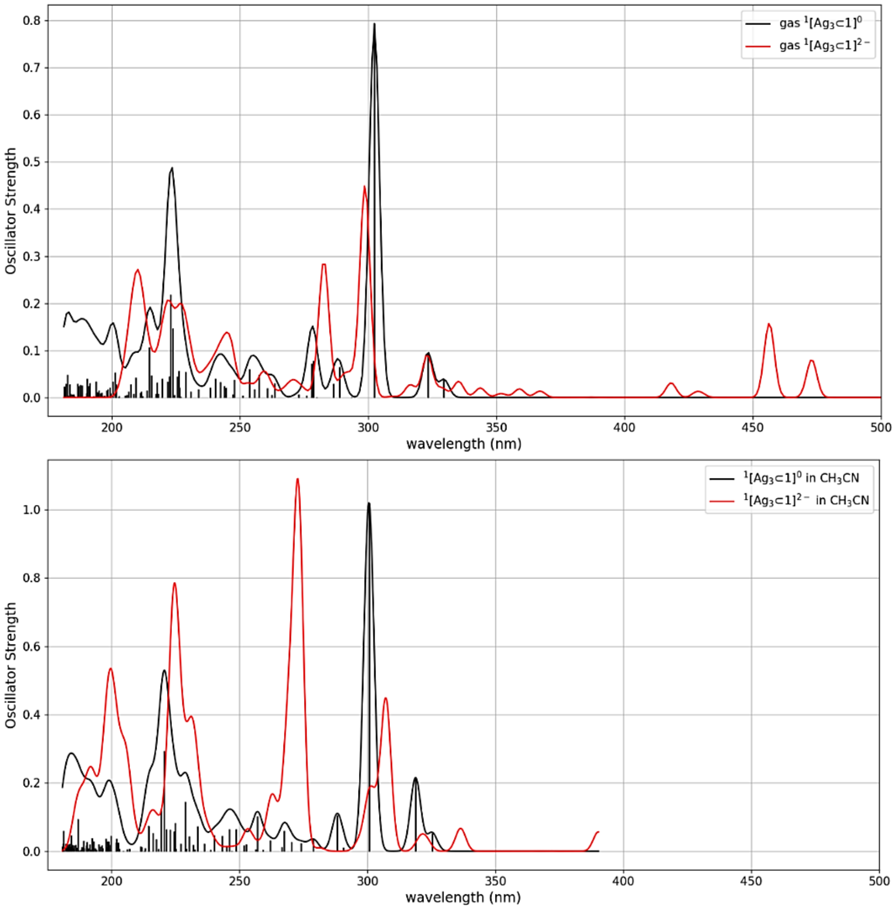

Figure S16. Comparisons of electronic excitations due to silver(I) and mixed-valence silver clusters in ^{1}[**Ag_3⊂1**]^{q} in the gas phase and in solution.

→ Despite differences in the shortwave UV region that may indicate mixed Ag valence, weak absorptions in the visible region appeared in the gas phase for ^{1}[**Ag_3⊂1**]$^{2-}$. In this case, the condition $Ag^{0} > Ag^{+}$ is met, so that colored compounds may be attributed to the presence of mixed-valence clusters.

F. Triplet-to-triplet transitions.

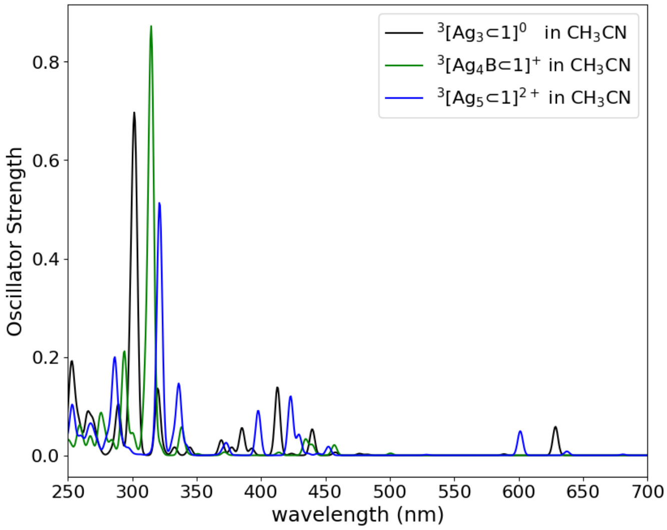

Figure S17. Effect of the Ag_n cluster size inside the cage **1** in the triplet-to-triplet spectra in solution.

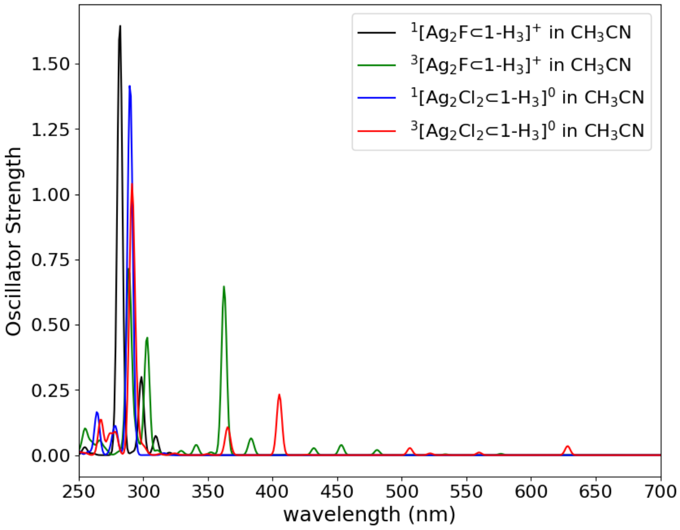

Figure S18. Comparisons of singlet-to-singlet and triplet-to-triple electronic transitions for silver(I) halides inside the cage **1-H**$_3$ in solution.

→ Spectra calculated via triplet-to-triplet electronic transitions account for light absorptions in the visible region.

G. Methodological aspects

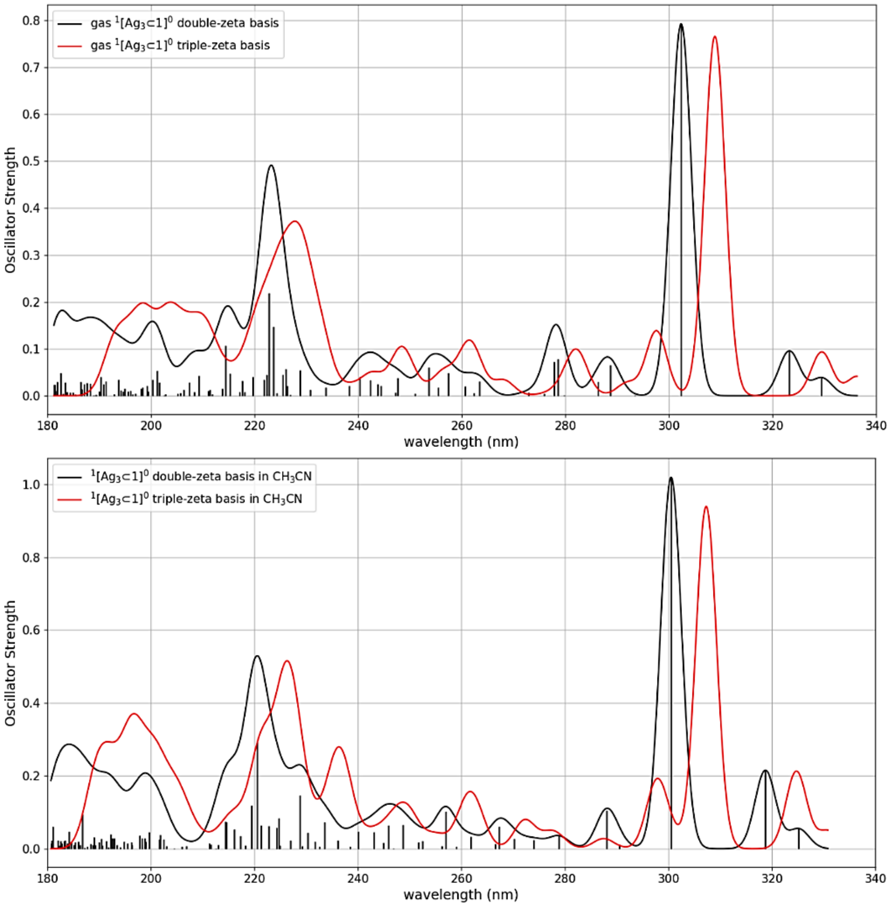

Figure S19. Comparisons of time-dependent DFT spectra of ^{1}[**Ag$_3$⊂1**]^0 calculated at different basis set size in the gas phase and in solution.

→ We compared the TDDFT spectra with those obtained with a larger basis set:

(*phase*) TD-*w*B97XD/6-311+G** ~ LANL2TZ // (*phase*) *w*B97XD/6-31G** ~ LANL2DZ

While the electronic transitions remain the same, the results suggest that excitation energies are red shifted, although within a small variation of less than 10 nm. Therefore, results using the double-zeta basis sets are similar to those with the triple zeta basis sets.

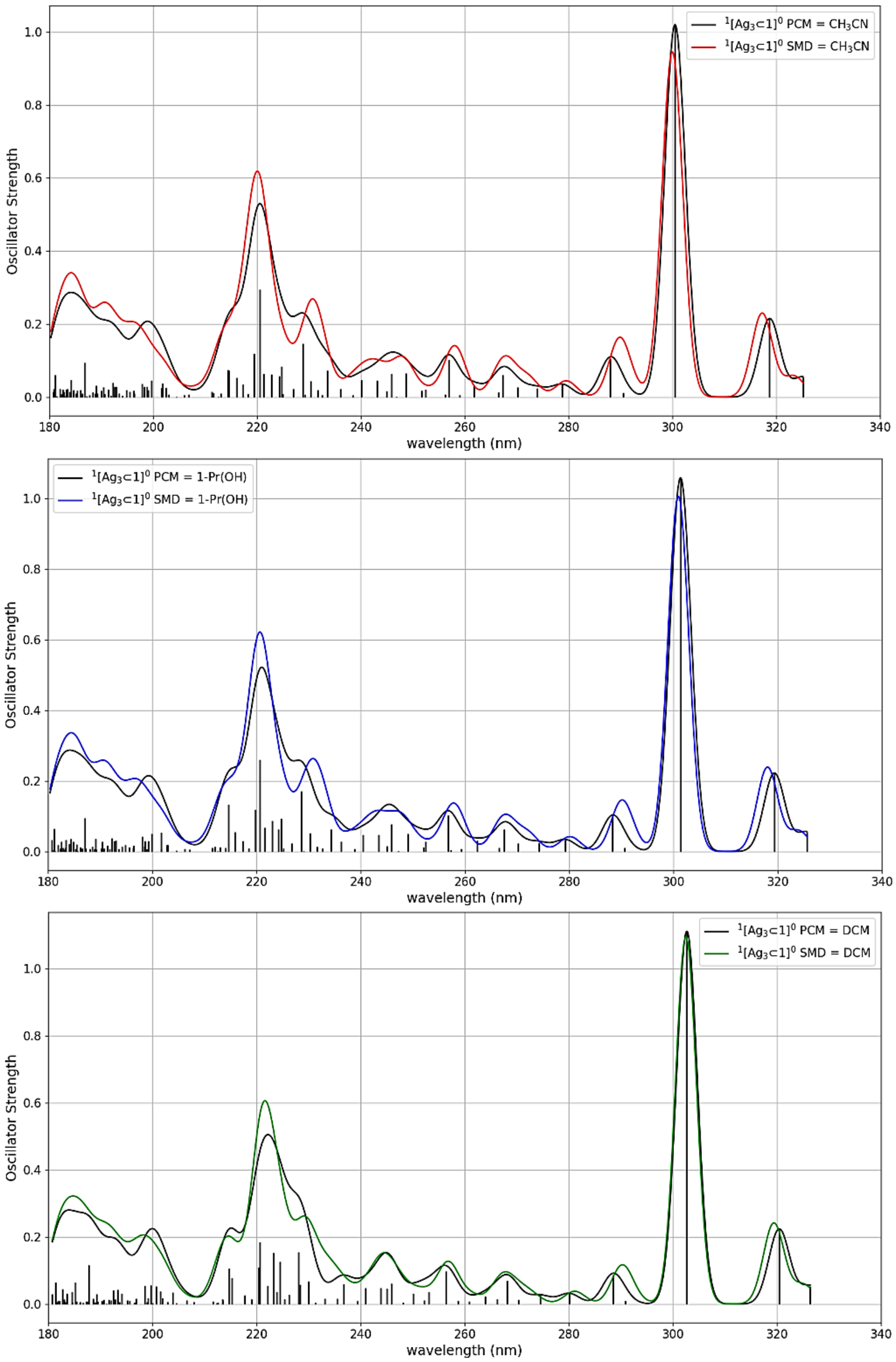

Figure S20. Comparisons of time-dependent DFT spectra of ^{1}[**Ag$_3$⊂1**]^0 calculated at different solvation models.

→ Results obtained with the PCM method are virtually identical to those calculated with the SMD approach.

Table S16. Selected Wiberg, Mayer and fuzzy bond order values for pyrrolide cages incorporating mixed-valence silver clusters.

bond	Wiberg index	Mayer index	Fuzzy index
**[Ag_3⊂1]$^{1-}$**			
Ag(1)-Ag(2)	0.25	0.10	0.25
Ag(1)-Ag(3)	0.33	0.14	0.36
Ag(2)-Ag(3)	0.28	0.09	0.26
**[Ag_3⊂1]$^{2-}$**			
Ag(1)-Ag(2)	0.24	0.07	0.23
Ag(1)-Ag(3)	0.30	0.11	0.32
Ag(2)-Ag(3)	0.29	0.08	0.27
**[Ag_3⊂1]$^{3-}$**			
Ag(1)-Ag(2)	0.66	0.57	0.69
Ag(1)-Ag(3)	0.62	0.53	0.65
Ag(2)-Ag(3)	0.66	0.49	0.71
**[Ag_4⊂1]^{0}**			
Ag(1)-Ag(2)	0.20	0.07	0.20
Ag(1)-Ag(3)	0.19	0.08	0.18
Ag(1)-Ag(4)	0.19	0.07	0.18
Ag(2)-Ag(3)	0.22	0.07	0.21
Ag(2)-Ag(4)	0.20	<0.05	0.18
Ag(3)-Ag(4)	0.23	0.07	0.21
**[Ag_4⊂1]$^{-1}$**			
Ag(1)-Ag(2)	0.43	0.36	0.47
Ag(1)-Ag(3)	0.43	0.36	0.46

Ag(1)-Ag(4)	0.43	0.34	0.47
Ag(2)-Ag(3)	0.47	0.34	0.51
Ag(2)-Ag(4)	0.46	0.33	0.51
Ag(3)-Ag(4)	0.47	0.32	0.52
**[Ag$_4$⊂1]$^{-2}$**			
Ag(1)-Ag(2)	0.49	0.47	0.55
Ag(1)-Ag(3)	0.30	0.15	0.27
Ag(1)-Ag(4)	0.37	0.21	0.37
Ag(2)-Ag(3)	0.45	0.26	0.48
Ag(2)-Ag(4)	0.45	0.31	0.45
Ag(3)-Ag(4)	0.34	0.16	0.32
**[Ag$_4$⊂1]$^{-3}$**			
Ag(1)-Ag(2)	0.19	0.08	0.17
Ag(1)-Ag(3)	0.22	0.08	0.22
Ag(1)-Ag(4)	0.19	0.08	0.17
Ag(2)-Ag(3)	0.20	0.06	0.17
Ag(2)-Ag(4)	0.22	0.07	0.20
Ag(3)-Ag(4)	0.21	0.06	0.19
**[Ag$_5$⊂1]$^{+1}$**			
Ag(1)-Ag(2)	0.28	0.17	0.34
Ag(1)-Ag(3)	0.06	<0.05	<0.05
Ag(1)-Ag(4)	0.26	0.18	0.31
Ag(1)-Ag(5)	0.29	0.17	0.36
Ag(2)-Ag(3)	0.28	0.17	0.34
Ag(2)-Ag(4)	0.21	0.09	0.22
Ag(2)-Ag(5)	0.21	0.09	0.22

Ag(3)-Ag(4)	0.29	0.17	0.36
Ag(3)-Ag(5)	0.26	0.18	0.31
Ag(4)-Ag(5)	0.24	0.13	0.30
**[Ag_5⊂1]^0**			
Ag(1)-Ag(2)	0.38	0.27	0.46
Ag(1)-Ag(3)	0.17	0.08	0.12
Ag(1)-Ag(4)	0.28	0.17	0.30
Ag(1)-Ag(5)	0.41	0.28	0.49
Ag(2)-Ag(3)	0.35	0.30	0.41
Ag(2)-Ag(4)	0.29	0.17	0.30
Ag(2)-Ag(5)	0.47	0.45	0.59
Ag(3)-Ag(4)	0.31	0.15	0.35
Ag(3)-Ag(5)	0.40	0.29	0.48
Ag(4)-Ag(5)	0.11	0.06	<0.05
**[Ag_5⊂1]$^{-1}$**			
Ag(1)-Ag(2)	0.33	0.19	0.36
Ag(1)-Ag(3)	0.08	<0.05	<0.05
Ag(1)-Ag(4)	0.32	0.15	0.35
Ag(1)-Ag(5)	0.31	0.14	0.34
Ag(2)-Ag(3)	0.33	0.19	0.36
Ag(2)-Ag(4)	0.39	0.36	0.45
Ag(2)-Ag(5)	0.39	0.36	0.45
Ag(3)-Ag(4)	0.31	0.14	0.34
Ag(3)-Ag(5)	0.32	0.15	0.35
Ag(4)-Ag(5)	0.27	0.13	0.26
**[Ag_5⊂1]$^{-2}$**			

Ag(1)-Ag(2)	0.30	0.19	0.31
Ag(1)-Ag(3)	0.10	0.09	<0.05
Ag(1)-Ag(4)	0.48	0.54	0.60
Ag(1)-Ag(5)	0.42	0.26	0.50
Ag(2)-Ag(3)	0.27	0.16	0.30
Ag(2)-Ag(4)	0.28	0.16	0.26
Ag(2)-Ag(5)	0.23	0.07	0.20
Ag(3)-Ag(4)	0.30	0.25	0.33
Ag(3)-Ag(5)	0.29	0.18	0.32
Ag(4)-Ag(5)	0.42	0.24	0.50
**$[Ag_5 \subset 1]^{-3}$**			
Ag(1)-Ag(2)	0.34	0.20	0.37
Ag(1)-Ag(3)	0.09	<0.05	<0.05
Ag(1)-Ag(4)	0.34	0.17	0.37
Ag(1)-Ag(5)	0.31	0.15	0.32
Ag(2)-Ag(3)	0.34	0.20	0.37
Ag(2)-Ag(4)	0.38	0.41	0.44
Ag(2)-Ag(5)	0.38	0.41	0.44
Ag(3)-Ag(4)	0.31	0.15	0.32
Ag(3)-Ag(5)	0.34	0.17	0.37
Ag(4)-Ag(5)	0.27	0.17	0.26
